# Metatranscriptomic and Metagenomic Analysis of Biological Diversity in Subglacial Lake Vostok (Antarctica)

**DOI:** 10.3390/biology9030055

**Published:** 2020-03-16

**Authors:** Colby Gura, Scott O. Rogers

**Affiliations:** Department of Biological Sciences, Bowling Green State University, Bowling Green, OH 43403, USA; cjgura@gmail.com

**Keywords:** Lake Vostok, subglacial lake, metatranscriptomic, metagenomic, marine, aquatic

## Abstract

A combined metatranscriptomic and metagenomic study of Vostok (Antarctica) ice core sections from glacial, basal, and lake water accretion ice yielded sequences that indicated a wide variety of species and possible conditions at the base of the glacier and in subglacial Lake Vostok. Few organisms were in common among the basal ice and accretion ice samples, suggesting little transmission of viable organisms from the basal ice meltwater into the lake water. Additionally, samples of accretion ice, each of which originated from water in several locations of the shallow embayment, exhibit only small amounts of mixing of species. The western-most portion of the embayment had very low numbers of organisms, likely due to biologically challenging conditions. Increasing numbers of organisms were found progressing from west to east, up to approximately 7 km into the embayment. At that point, the numbers of unique sequences and sequence reads from thermophilic, thermotolerant, psychrophilic, and psychrotolerant organisms increased dramatically, as did sequences from alkaliphilic, alkalitolerant, acidophilic, and acidotolerant sequences. The number of unique and total sequences were positively associated with increases in concentrations of Na^+^, Ca^2+^, Mg^2+^, SO_4_^2−^, Cl^−^, total amino acids, and non-purgeable organic carbon. The numbers of unique sequences from organisms reported from soil, sediment, ice, aquatic, marine, animal, and plant (probably pollen) sources also peaked in this region, suggesting that this was the most biologically active region. The confluence of the high numbers of organisms, physiologies, and metabolic capabilities suggests the presence of energy and nutrient sources in the eastern half of the embayment. Data from the main basin suggested a cold oligotrophic environment containing fewer organisms. In addition to bacteria, both the basal ice and accretion ice contained sequences from a diverse assemblage of eukaryotes, as well as from bacteria that are known to be associated with multicellular eukaryotes.

## 1. Introduction

**Figure 1:**
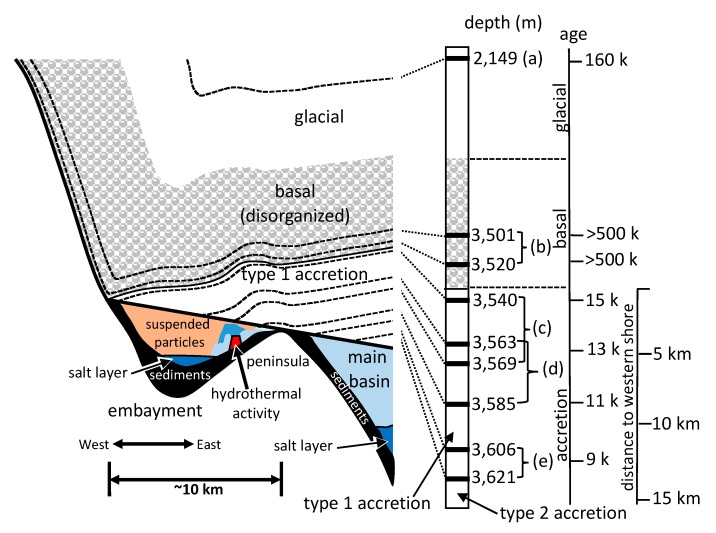
Profile model of the portion of Lake Vostok (left half) that is the focus of this research, based on the data from this research and elsewhere [1,2]. Ice core sections used in this research are shown (black bars, middle right), as part of the Vostok ice core. For both parts of the figure, the upper white portions are glacial ice (3310 m) that is in layers (right). The next 228 m is basal ice (shaded with gray ellipses), type 1 accretion ice (shaded in solid gray), and type 2 accretion ice (white). The ice core samples used for this research are listed (a–e). Approximate ages of the ice and approximate distances from the western shore (according to maps from ref. [1,2,6,7]) are on the far right. Possible hydrothermal activity is indicated on the eastern side of the embayment (red trapezoid).

Subglacial Lake Vostok is an extreme environment that appears to support a diverse ecosystem concentrated in a shallow embayment on the southwestern corner of the lake [1,2]. Of the nearly 400 subglacial lakes that have been reported in Antarctica, Lake Vostok is by far the largest [3]. Located approximately 1300 km from the South Pole in East Antarctica, it covers an area of 12,500 km^2^, with a volume of 5400 km^3^, and a maximum depth of 1067 m, This makes it one of the largest, most voluminous, and deepest lakes on Earth. However, it is covered by 4 km of glacial ice, has an estimated temperature that hovers just below 0 °C, and has been continuously ice covered for approximately 14–15 million years [4,5,6]. Additionally, its surface lies 200 m below sea level, resting in a graben of a rift valley [7]. The overriding glacier blocks all light from reaching the lake and exerts approximately 35 megapascals of pressure on the lake [8]. It delivers sediment and glacial flour from the action of the glacial surfaces grinding away portions of bedrock (Figure 1), as well as living and dead organisms, minerals, and atmospheric gases [1,2,5,8,9]. Because of its low elevation, at one time it may have been part of the surrounding Southern Ocean [1]. There is evidence for hydrothermal activity in the region of a shallow embayment on the southwest corner of Lake Vostok [1,2,9,10]. In addition to adding energy, it may be delivering several chemicals that are being utilized and metabolized by organisms living in the lake. The combination of temperature extremes, high pressure, frictional forces, melting, freezing, and ion gradients, make Lake Vostok a challenging environment for organisms.

The glacier contains several distinct types of ice that have been formed by different processes (Figure 1). At Vostok Station on the southeast corner of the lake, the upper 3310 m of ice is meteoric (the result of snowfall) and exists in layers, having been deposited over a period of 420,000 years [4]. The 228 m of ice below this, called basal ice, is also meteoric in origin, but it has been disturbed due to its interaction with the bedrock below. Although some of it is estimated to be more than one million years old, it cannot be accurately dated because of its deformation [11]. Below the basal ice is a 231 m layer of ice that differs from the glacial and basal ice. It consists of accretion ice that is lake water frozen to the bottom of the glacier. Because the glacier is flowing across the lake, the accretion ice holds a positional and temporal ice record of the various regions of the lake surface. This layer is thinnest where the glacier enters the lake on its western shore, and thickest where the glacier exits the lake on its eastern shore, close to where the Vostok Station ice core drilling operation is located. Furthermore, most of the ice that accreted over the western section of the lake (within a shallow embayment) contains large amounts of fine particulate matter (termed type 1 accretion ice), while most of the ice that has accreted over most of the main basin of the lake is crystal clear, containing almost no particulates at all (termed type 2 accretion ice). This natural sampling of the lake water has allowed study of Lake Vostok by examining ice core sections of the accretion ice without contaminating the lake itself. Research has been conducted on the geochemistry, geological, and biological inclusions in the accretion ice, which has provided information about the conditions in the lake [1,2,5,8,9].

Initial hypotheses proposed that Lake Vostok was sterile due to the extreme and oligotrophic conditions. However, as information from geochemical, biological, and biochemical studies has accumulated, the results have suggested the existence of a complex biological ecosystem in the lake waters and sediments [1,2,9,10,12,13,14,15]. Furthermore, the variety of conditions in the shallow embayment appear to have led to the formation of several ecological zones. Most of the biological activity, as measured from the accretion ice collected from the Vostok 5G ice core, is concentrated within or near the shallow embayment, which is approximately 10 km wide in the southwest corner of the lake. While the sources of some of the nutrients, biological molecules, and ions have been determined, questions remain regarding the sources of the nucleic acids, organic carbon, and viable organisms that have been recovered from the accretion ice. The metatranscriptomic and metagenomic research described here was initiated to determine the types of microbes being delivered to Lake Vostok by the glacier, and to compare the results with the community of organisms found in the accretion ice from the shallow embayment and western portion of the southern main basin.

## 2. Materials and Methods

### 2.1. Acquisition and Processing of Ice Core Sections

Vostok 5G ice core sections from each of four regions were obtained from the NSF-ICL (National Science Foundation - Ice Core Laboratory, Lakewood, Colorado): glacial ice (2149 m), basal ice (3501 m and 3520 m), ice from the west region of the shallow embayment (3450; type 1 accretion ice), ice from the middle of the embayment (3569 m; type 1 ice), and ice from the east region of the embayment (3585; type 2 ice). Four Vostok 5G ice core sections (3563, 3585, 3306, and 3621 m) were analyzed in a previous study [1,2]. The results are compared in this research study. The ice core sections had been drilled and extracted at Vostok Station, Antarctica (78.28°S, 106.48°E) by a Russian drilling crew in collaboration with Russian, US, French, British, and other science teams, from the glacier overlying Lake Vostok. The core sections were shipped frozen to our laboratory at Bowling Green State University, Bowling Green, Ohio; and were stored at −20 °C. Sections were chosen based upon desired depths and absence of cracks. The selected sections were cut into lengths of 6–16 cm and warmed at 4 °C for 30 min prior to melting. All sterilization and melting procedures were conducted in a sterile laminar flow biosafety hood. The hood was inside a positive pressure room supplied with HEPA (high efficiency particulate air) filtered air. All lab bench and hood surfaces were sterilized with a 5.25% sodium hypochlorite solution, followed by 70% ethanol, and exposed to UV irradiation for 1 hour prior to sterilization and melting of ice core sections. Inside the sterile laminar flow hood, the ice core sections were completely immersed in a 5.25% sodium hypochlorite solution (chilled to 4 °C) for 10 sec followed by 3 sequential rinses with 800 mL of autoclaved sterile reverse osmosis (RO) water (4 °C, 18.2 MΩ, <1 parts per billion (ppb) total organic carbon (toc)). Each treated ice core section was then melted at room temperature inside the hood using a sterile funnel, collecting aliquots of between 25 and 50 mL in sterile 50 mL screw cap tubes, placed on ice (frozen sterile RO water). In order to assure a sufficient amount of DNA and RNA, meltwater from the 3501 and 3520 m sections were mixed, and meltwater from the 3540 and 3569 m sections were mixed, to obtain a total volume of at least 100 mL each. Only 61.5 mL was available from the 3585 m section. The contents of five samples (2149 m; 3501 + 3520; 3540 + 3569; 3585; and a sterile RO water control) were then concentrated by ultracentrifugation at 100,000× g for 14 h, followed by resuspension in 100 uL of sterile RO water, and purified using QIAamp MinElute virus spin columns (QIAGEN, Venlo, Netherlands), avoiding the use of the carrier RNA supplied in the kit (which would introduce contaminating sequences). The nucleic acids were purified using TRIzol reagent (Thermo Fisher Scientific, Inc., Columbus, OH) for the RNAs and CTAB (cetyltrimethylammonium bromide) for the DNAs [1,2,16].

### 2.2. cDNA Synthesis

The isolated RNA was used to synthesize cDNA employing a SuperScript II kit (Invitrogen, Carlsbad, California) according to the manufacturer’s instructions. First, random hexamer primers were added to each 5 μL (RNA plus DNA) sample to a concentration of 6.5 ng/μL. Each sample was then mixed gently, incubated at 70 °C for 10 min, and then chilled on ice. The first strand synthesis consisted of 33.3 mM Tris-HCl (pH 8.3), 50 mM KCl, 2 mM MgCl_2_, 6 mM DTT (dithoithreitol), 330 μM dNTPs (82 μM of each dATP, dCTP, dGTP, dTTP), and 100 units of SuperScript II reverse transcriptase (final volume of 15.5 μL) for each sample; incubated at 37 °C for 90 min and then placed on ice. Second strand synthesis consisted of 25 mM Tris-HCl (pH 6.9), 93 mM KCl, 4.6 mM MgCl_2_, 140 μM β-NAD^+^, 9.3 mM (NH_4_)_2_SO_4_, 0.5 mM dNTP mix (as above), 1.2 mM DTT, 5 U *Escheria coli* DNA ligase, 20 U *E. coli* DNA polymerase I, and 1 U *E. coli* RNase H (in a total of 80 µL); incubated for 2.5 h at 15 °C. Next, 5 U T4 DNA polymerase was added to each sample and incubated at 15 °C for 7 min, and then placed on ice. The reaction was stopped by the addition of EDTA to a concentration of 30 mM. Then, each sample was subjected to chloroform/isoamyl alcohol (24:1) treatments, centrifuged at 14,000× g for 2 min to separate the layers, and the nucleic acids (aqueous phase) were precipitated in 0.5 M NaCl and 80% ethanol. Precipitation was performed at −20 °C for 15 min. After centrifugation at 14,000× g for 20 min, the pellets were washed with -20 °C 80% ethanol, and centrifuged at 14,000× g for 2 min. After decanting, the pellets were dried in a vacuum centrifuge drying (Eppendorf Vacufuge, Hauppauge, NY) at 45 °C for 10 min, and then were rehydrated in 9 μL of DEPC-treated water. These were mixed with the corresponding DNAs. 

### 2.3. Adapter Ligation and Amplification

Adapter sequences were added to the ends of each of the DNAs and cDNAs in order to amplify them in subsequent steps. They were added in the following reaction: 66 mM Tris-HCl (pH 7.6), 10 mM MgCl_2_, 1 mM ATP, 100 pmols *Eco*RI (*Not*I) adapters (AATTCGCGGCCGCGTCGAC), 14 mM DTT, and 2.5 U T4 DNA ligase (total volume 25 µL); incubated at 15 °C for 16 h, and heated to 70 °C to stop the reaction. Next, 15 U T4 polynucleotide kinase were added, and incubated at 37 °C for 30 min. To stop the reaction, the samples were heated to 70 °C. Sephracyl size fractionation columns (supplied with the Superscript II kit) were rinsed with TNE (10 mM Tris (pH 7.5), 0.1 mM EDTA, 25 mM NaCl), and used to collect 23 fractions (35 µL each), each of which was subjected to PCR (polymerase chain reaction) amplification (20 mM Tris (pH 8.3), 10 mM KCl, 10 mM (NH_4_)_2_SO_4_, 0.8 mM dNTPs, 2 mM MgCl_2_, 0.4% Triton X-100, 10 mg bovine serum albumen, 1 U *Taq* DNA polymerase (AmpliTaq Gold DNA polymerase, Applied Biosystems, Foster City, California), 1 μM *Eco*RI (*Not*I) primer (AATTCGCGGCCGCGTCGA), and 5 µL of each DNA/cDNA sample, in a total volume to 25 µL. The thermocycling program was 94 °C for 2 min followed by 45 cycles of 94 °C for 1 min, 55 °C for 2 min, 0.1 °C/sec ramp to 72 °C, and 72 °C for 2 min. Once the cycles were finished, the samples were incubated at 72 °C for 10 min before being held at 4 °C. All amplified fractions were subjected to electrophoresis on 2% agarose gels (Type 1, low EEO, Sigma-Aldrich, St Louis, Missouri) at 5 V/cm in TBE (89 mM Tris, 89 mM borate, 2 mM EDTA), 0.5 μg/mL ethidium bromide, and visualized with UV irradiation to determine concentrations and size distributions. 

### 2.4. EcoRI/NotI MID Primer Polymerization and Purification

Fractions 3–13 from each sample were used for further processing, based on having DNA distributions between approximately 100 and 1000 bp. Next, a total of five pairs of primers were synthesized. Each contained one of a set of multiplex identifier (MID) sequences on the 5’ end of the primers (MID2A-ACGCTCGACA, for the 2149 m sample; MID4A-AGCACTGTAG, for the 3501 + 3520 m sample; MID5A-ATCAGACACG, for the 3540 + 3569 m sample; MID6A-ATATCGCGAG, for the 3585 m sample; MID10A-TCTCTATGCG, for the water control), and the EcoRI/NotI sequences (AATTCGCGGCCGCGTCGAC) on their 3’ ends. The primers were used in PCR reactions (as above) with 1 μM corresponding to each MID *Eco*RI primer pair, and 1 µL of previously amplified DNA/cDNA samples in a total volume to 25 µL. The samples were purified using a QIAquick PCR purification kit (QIAGEN, Venlo, Netherlands), and quantified on 2% agarose gels (as above). 

### 2.5. Ion Torrent Adapter Addition

Ion Torrent adapters were ligated to the ends of the amplicons (from the previous step), using an NEBNext for Ion Torrent Library Preparation Kit (New England Biolabs, Ipswich, MA). Each sample was prepared as follows: 1 ug DNA sample, end repair buffer (50 mM Tris-HCl (pH 7.5), 10 mM MgCl_2_, 10 mM DTT, 1 mM ATP, 0.4 mM dATP, 0.4 mM dCTP, 0.4 mM dGTP, 0.4 mM dTTP), end repair enzyme mix (NEBNext system, Klenow fragment and T4 DNA polymerase). Samples were then incubated for 20 min at 25 °C, then 10 min at 70 °C, and held at 4 °C. Ligation of the Ion Torrent adapters was accomplished by adding NEB T4 ligase buffer for Ion Torrent, Ion Torrent Primers (A-CCATCTCATCCCTGCGTGTCTCCGACTCAG; and P1-CCTCTCTATGGGCAGTCGGTGAT), NEBNext Bst 2.0 WarmStart DNA polymerase, and T4 DNA ligase (in a total volume of 100 µL). This was incubated for 15 min at 25 °C, then 5 min at 65 °C, and finally held at 4 °C. Each was precipitated with 0.5 M NaCl and 80% ethanol at −20 °C for 1 h. The nucleic acids were pelleted by centrifugation in a microfuge, and dried in a vacuum centrifuge (Eppendorf Vacufuge, Hauppauge, NY) at 45 °C for 10 min. Each was rehydrated in 50 µL 1 mM Tris (pH 7.5), 0.1 mM EDTA.

### 2.6. Amplification with Ion Torrent MID primers

A reaction for each sample was performed with 20 µL of the products from the step above, 1 uM of the corresponding pair of primers (form of primers was A-M-E, or P1-M-E, where A and P1 are the Ion Torrent-specific regions, M is the MID sequence for each of the five samples (listed above), and E is the EcoRI/NotI sequence), and 50 µL of the NEBNext Q5 hot Start HiFi PCR master mix (total of 100 µL). The thermocycler program was 98 °C for 30 sec followed by 15 cycles of 98 °C for 10 sec, 58 °C for 30 sec, 65 °C for 30 sec and once complete, the samples were incubated at 65 °C for 5 minutes, with a 4 °C hold. Once polymerization was complete the samples were purified with QIAquick columns using the previous procedure. Each was rehydrated in 1 mM Tris (pH 7.5), 0.1 mM EDTA, and quantified by 2% agarose gel electrophoresis (as above).

### 2.7. Ion Torrent Sample Preparation

Two samples were prepared for sequencing at the University of Pennsylvania Core Sequencing Lab (Philadelphia, PA). Sample one consisted of a mix of 150 ng of product from the negative control (sterile RO water), 150 ng from the 2149 m ice core section sample, and 150 ng from the 3501 + 3520 m ice core section samples, for a total of 450 ng in a volume of 50 µL. The second sample consisted of a mixture of 225 ng of product from the 3540 + 3569 m ice core section samples, and 225 ng from the 3585 m ice core section sample, for a total of 450 ng in a final volume of 50 µL. 

### 2.8. Sequence Curation and BLAST Searches

The sequences were received from the University of Pennsylvania Core Sequencing Lab as two files in FASTQ format and converted to FASTA format using Biopython (Open Bioinformatics Foundation). Only high-quality reads were used. The files were then divided in silico into their original five groups based upon the MID sequences within the primers (see Supplemental File S1, Biopython scripts). Next, the primer sequences were clipped from all of the reads in silico for each of the five groups. Finally, any reads less than 50 base pairs were removed.

To achieve efficient and accurate organism affiliations from the sequence data, stand-alone BLAST+ and the NCBI BLAST nucleotide and taxonomy databases were downloaded. BLAST searches were performed locally on each of the prepared files using the command: “blastn -db nt -query *input file* -out *output file.csv* -outfmt “10 qseqid sacc sgi length sstart send pident evalue staxid sskingdom ssciname” -max_target_seqs 20”. In addition to the glacial (2149 m), basal (3501 + 3520 m), west embayment (3540 + 3569 m), and east embayment (3585 m) data sets, sequence data from our previous research, including data from the middle of the embayment (3563 + 3585 m) and the western region of the southern main basin (3606 + 3621 m) samples (Figure 1; References [1,2]) were reanalyzed using the same procedures. For all final comparisons, cut-off values of ≥97% identity and e-value < 10^−6^ were used. Approximately 33% of the sequences from each ice core section depth failed to return any similar sequences at any identity level during the database searches.

Using custom Biopython scripts (Supplemental File S1), sequences that were found in the negative control were removed from the other datasets based upon sequences with a percent identity ≥97% for the same GI numbers. Enumeration of read frequencies for each species were calculated. For most analyses, duplicate GI numbers were eliminated by keeping the GI number with the lowest e-value and highest percent identity. Then, the NCBI sequence that the query sequence matched during the BLAST search was retrieved and put into three categories: rRNA, mRNA, and other sequences. The rRNA genes were primarily used to confirm taxonomic affiliation. The mRNA genes were used to determine taxonomic affiliations and to analyze metabolic pathways. The phyla corresponding to each genus and species were determined and associated with each sequence. Shannon–Weaver diversity analyses were performed for the basal and accretion ice samples.

### 2.9. Ecology and Physiology

The ecological niches and metabolic characteristics were retrieved for each species and/or strain (that had been determined by the BLAST results), by searching NCBI information, publications, and other online resources. Species and/or strains were used exclusively to deduce ecological and physiological affinities. Determinations were made only when they were unambiguous. Organisms were then categorized into groups based upon gene identities (from BLAST), ecology, source, physiology, metabolic capabilities, and other notable species characteristics. Additionally, the data were used to compile information on metabolic pathways, such as nitrogen cycling and carbon fixation. 

### 2.10. Comparison of Data

Metatranscriptomic and metagenomic data from refs. [1,2] were retrieved for comparison to contemporary data. In those studies, the data pertaining to glacial depths of 3563 m and 3585 m, respectively corresponding to the middle and east embayment (type 1 and type 2 accretion ice, respectively), had been combined into one sample (named V5 in refs. [1,2]). Data from glacial depths of 3606 m and 3621 m, both corresponding to the western region of the southern main basin (type 1 and type 2 accretion ice, respectively), were combined together (named V6 in refs. [1,2]). Those samples were originally analyzed in a similar fashion to those being analyzed here. Sequencing data from the 2013 studies and the data presented here were compared to determine the overlaps in taxa within the glacial, basal, and accretion ice using custom Biopython script (Supplementary File S1). The matching organisms along with their corresponding phyla and domains were saved in Excel files for analyses. 

Associations between the number of species (determined here and from refs. [1,2]) and concentrations of Na^+^, K^+^, Ca^2+^, Mg^2+^, Cl^−^, SO_4_^2−^, non-purgeable organic carbon (NPOC), and total amino acids (from ref. [9]) were statistically examined. A multi-level mixed-effects-negative-binomial regression model was used to account for the overdispersion in the dependent variable (number of species) and the repeated sampling in the different habitats (soil, freshwater, marine, ice, animal-associated, and plant-associated). The explanatory variables (Na^+^, K^+^, Ca^2+^, Mg^2+^, Cl^−^, SO_4_^2−^, NPOC, and amino acids) were modelled as fixed effects and the type of habitat was modeled as a random intercept. Alpha was set at 0.05, 2-tailed.

## 3. Results and Discussion

### 3.1. Summary of Results

National Center for Biotechnology Information (NCBI) sequence accession numbers are as follows: 2149 m - SAMN12175388; 3501 + 3520 m - SAMN12175389; 3540 + 3569 m - SAMN12175390; 3585 m - SAMN12175391. The BioProject that contains information related to the samples listed above located at the NCBI sequence read archive can be retrieved using the accession number PRJNA552298. The amount of sequence data for each sample was as follows: 2149 m (glacial) = 4571 bp; 3501 + 3520 m (basal) = 46,421,725 bp; 3540 + 3569 m (western embayment, type 1 accretion ice) = 50,689,858 bp; and 3585 m (eastern shallow embayment, type 2 accretion ice) = 71,241,841 bp. 

**Figure 2:**
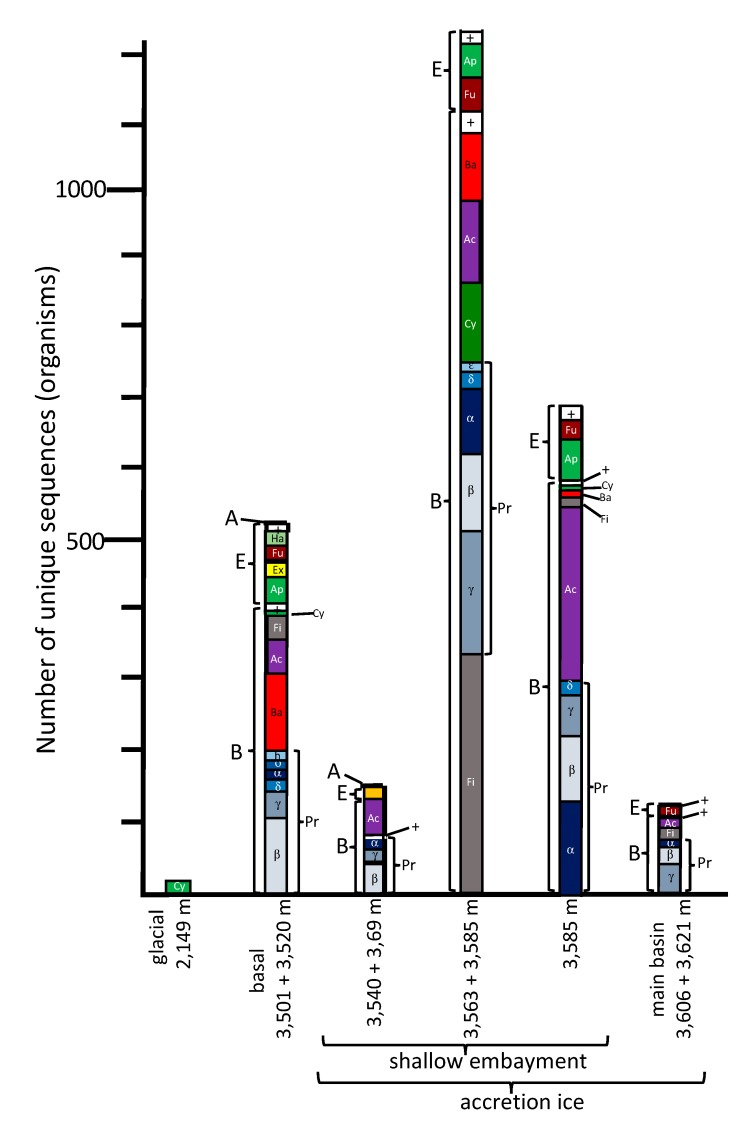
Comparisons of the total numbers and taxonomic affinities of sequences from each of the ice core samples. Abbreviations: Domains—A = Archaea; B = Bacteria; E = Eukarya. Other taxonomic designations: Ac = Actinobacteria; Ap = Archaeplastida; Ba = Bacteroidetes; Ch = Chlorophyta; Cy = Cyanobacteria; Ex = Excavata; Fi = Firmicutes; Fu = Fungi; Ha = Haptophyta; Pr = Proteobacteria (α—Alphaproteobacteria, β—Betaproteobacteria, δ—Deltaproteobacteria, ε—Epsilonproteobacteria, γ—Gammaproteobacteria, h—Hydrogenophilalia, o—Oligoflexia); + = a mixture of small numbers of other taxa.

**Figure 3:**
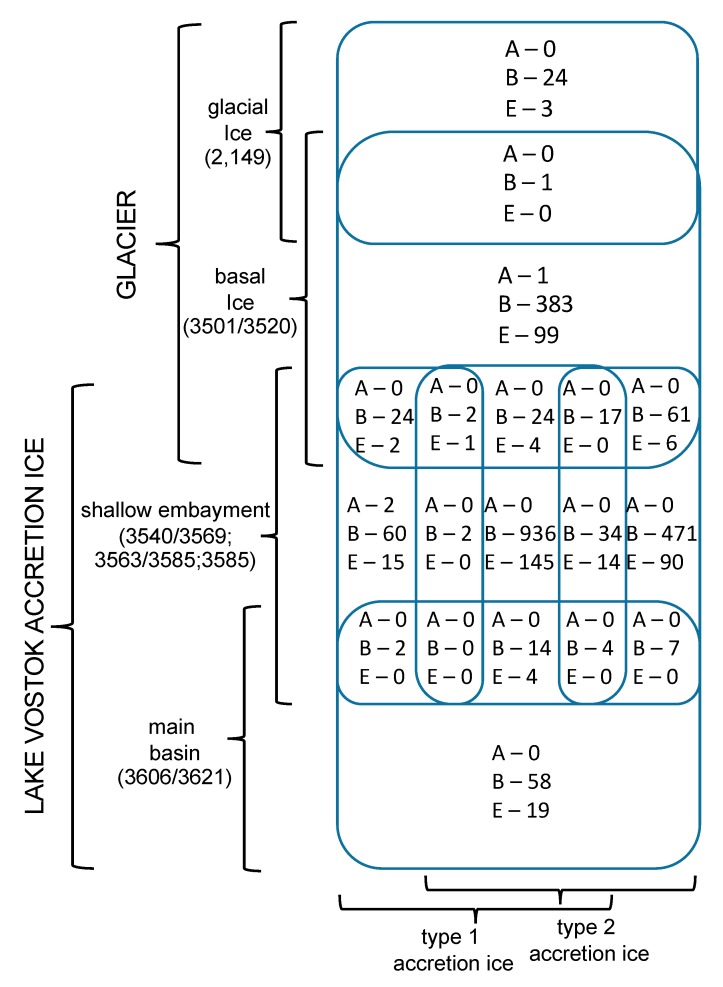
Venn diagram indicating the numbers of unique sequences in each core sample, and the numbers of sequences that are found in adjacent samples (overlapping rounded rectangles). Domain abbreviations are as in Figure 2.

For most of the analyses, all duplicates were removed, such that only unique sequences were considered in the characterizations and comparisons (Tables S2–S7). The only exceptions were for the diversity index calculations, where enumeration of the number of times the same accession numbers appeared (i.e., reads) was used (Table S8). Sequences from previous research (middle embayment, sections 3563 + 3585 m, representing type 1 and type 2 accretion ice; and main basin, which included sections 3606 + 3621 m, representing type 1 and type 2 accretion ice; [1,2]) were reanalyzed for consistency, and compared with the analyses of the data generated in this research. The total number of unique species represented by the sequence data, using a cut-off value of ≥97% and an e-value of 10^−6^ from all samples, was 2644 (Figure 2). Of these, 513 were from the glacial and basal ice core sections, and the remainder (2131) were from the accretion ice, with most of those (2038) being from the shallow embayment accretion ice. The remaining 93 were from the main basin accretion ice. Sequences from bacterial and eukaryotic species were present in all of the samples (Figure 2). Overall, more than 75% of the unique sequences were from bacteria, and almost 25% were from Eukarya. However, in some samples, up to 90% of the unique sequences were from Bacteria, and as low as 10% were from Eukarya (e.g., 3563 + 3585 m sample from the middle of the shallow embayment). Only 0.2% among all samples were from Archaea (*Halorubrum trapanicum*, *Halobacterium salinarum* R1, and *Halobacterium salinarum* NRC-1, three species adapted to high-salt aquatic and marine environments), and they were limited to the basal ice (3501 + 3520 m) and the western and middle sections of the shallow embayment (3540 + 3569, and 3563 + 3585 m samples, respectively). Ice from this region was composed primarily of type 1 accretion ice. The number of unique and shared sequences for each sample indicated that each sample contained a majority of sequences that were unique to that sample (Figure 3). Specifically, there was only one sequence in common among the glacial (2149 m sample) and basal ice (3501 + 3520 m sample). Additionally, only low numbers of sequences were in common among the basal and accretion ice samples, indicating that few organisms from the basal ice are present in the lake water. Similarly, only a small amount of mixing is evident from the ice samples representing the embayment and main lake basin (Figure 3). This might be partially explained by the fact that the embayment is approximately 10 km wide, and a peninsula partially separates it from the main basin in the portion of the accretion ice that was sampled. Additionally, there is a temporal component (Figure 1). The accretion ice that was sampled from the western section of the embayment was formed approximately 15,000 years ago, while the ice that formed over the eastern portion of the embayment formed several thousand years later, and the ice over the western main basin formed a few thousand years after that.

### 3.2. Glacial Ice (2149 m Sample)

The low quantity of sequence data (12 unique sequences at ≥97% identity) from the 2149 m sample is consistent with other studies that have reported low concentrations of cells at this depth of the glacier [9,12]. All of the sequences found here were from uncultured cyanobacteria. Sequences from the same uncultured cyanobacteria also were present at low levels among the other samples (Figure 2), including the basal ice (3510 + 3520 m), and samples from the shallow embayment (3540 + 3569, 3563 + 3585, and 3585 m). Whether or not these were contaminants from the drilling process could not be completely ruled out. However, several other members of Cyanobacteria were found in the 3563 + 3585 m sample. Because of the lack of sunlight, the cells may be dead, although viable Cyanobacteria have been isolated from deep (and dark) permafrost, and they can survive indefinitely as heterotrophs without sunlight [17]. Therefore, it is possible that many of these cells are living. No members of Cyanobacteria were detected in the main basin sample (3606 + 3621 m), or in the negative control.

### 3.3. Basal Ice (3501 + 3520 m Sample)

The basal ice (3501 + 3520 m sample; >500,000 years old) contained a diverse set of organisms (Shannon–Weaver diversity index = 4.52, evenness = 0.48), including large numbers of Bacteria (407 unique sequences at ≥97% identity) and Eukarya (103 unique sequences), plus two of the Archaea (*H. salinarum* R1 and *H. salinarum* NRC-1) mentioned above (Figure 2; Tables S2 and S8). The Bacteria were primarily members of Proteobacteria (nearly 200 species, of which 50% were Betaproteobacteria, 16% were Gammaproteobacteria, and the remainder was a mixture of Alphaproteobacteria, Deltaproteobacteria, Epsilonproteobacteria; and single species each in Hydrogenophilalia and Oligoflexia) and Bacteroidetes (slightly more than 100 species), with fewer from Actinobacteria (50 species), Firmicutes (40 species), and a variety of organisms from other phyla. The eukaryotes (103 species) were within the taxa Alveolata, Animalia, Archaeplastida, Excavata, Fungi, and Haptophyta, in roughly equal proportions. 

**Figure 4:**
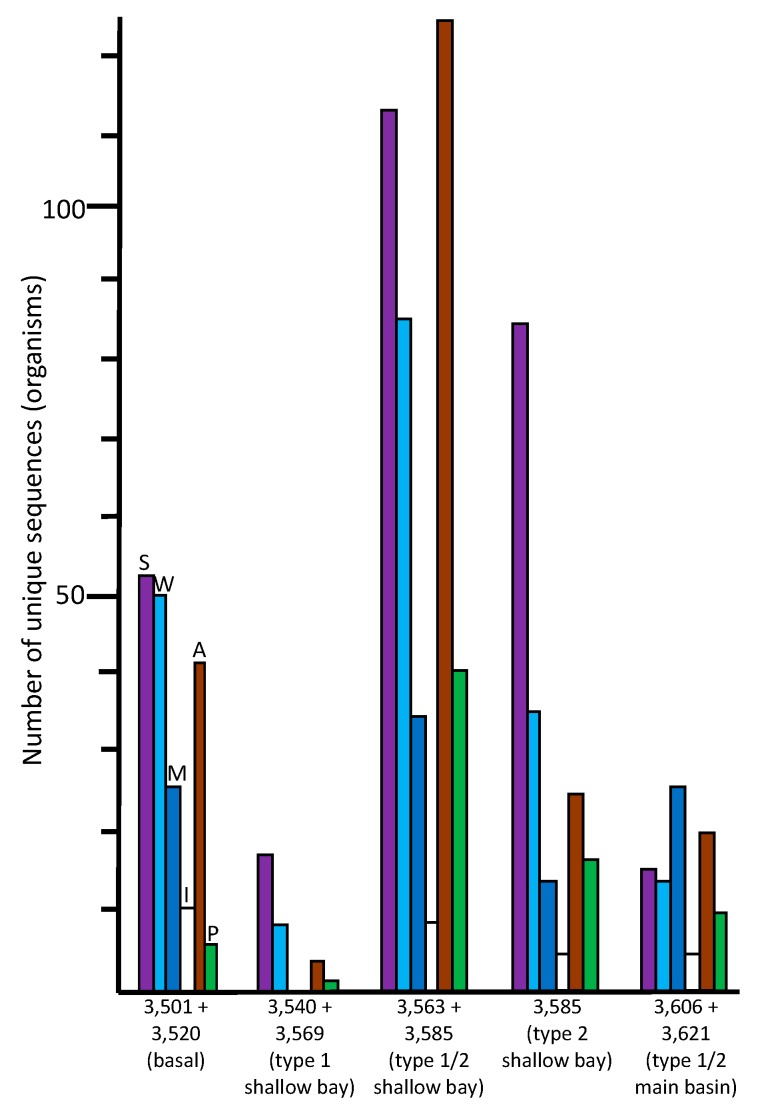
Numbers of unique sequences in the ice core samples that matched sequences from organisms in the NCBI database (≥97% identity, e-value <10^−6^) that had been isolated from soil/sediment (S, violet), aquatic/freshwater (W, light blue), marine/saltwater (M, dark blue), permafrost/ice (I, white), animal (A, brown), or plant (P, green) sources.

**Figure 5:**
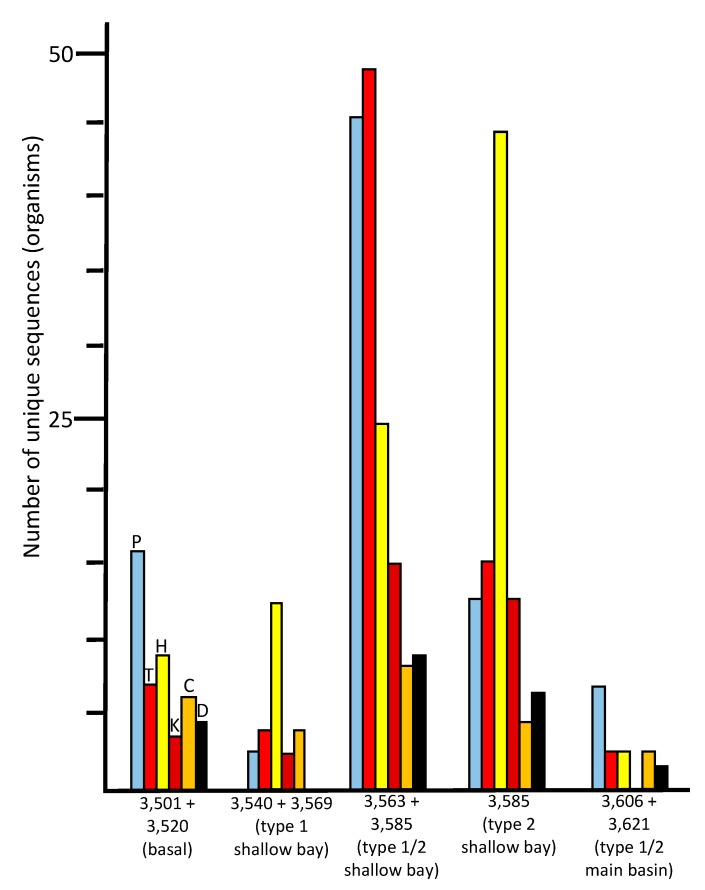
Numbers of unique sequences in the ice core samples that matched sequences from organisms in the NCBI database (≥97% identity, e-value <10^−6^) that are known to be psychrophilic/psychrotolerant (P, sky blue), thermophilic/thermotolerant (T, red), halophilic/halotolerant (H, yellow), alkaliphilic/alkalitolerant (K, brick red), acidophilic/acidotolerant (C, orange), or desiccation tolerant (D, black).

The species indicated in the basal ice have been previously described from soil, aquatic, marine, and ice environments (Figure 4). These results are consistent with a glacier/bedrock interface, which likely includes subglacial streams, lakes, ponds, and other features at higher elevations. A large number of the species have been described as associated with animals (Figure 4; Tables S2, S7 and S8), and a few that are associated with plants. The highest number of sequences were closest to psychrophilic and psychrotolerant species, although there were some that were closest to thermophilic and thermotolerant species, as well as a few sequences closest to halophilic/halotolerant, alkaliphilic/alkalitolerant, acidophilic/acidotolerant, and desiccation-resistant species (Figure 5). This suggests a complex ecosystem in the basal ice upstream from the lake.

### 3.4. Western Section of Embayment (3540 + 3569 m Sample–Type 1 Accretion Ice)

The number of unique species (based on sequence data) in the 3540 + 3569 m sample was much lower (130 at ≥97% identity) than those found in the basal ice sample (513) and the adjacent deeper middle embayment accretion ice sample, 3563 + 3585 m (1214). This portion of the accretion ice has large concentrations of suspended particles (type 1 accretion ice; Figure 1), indicating an area of turbid water. It is also within or close to the grounding line of the glacier. Few species were in common with those from the basal ice core section (Figure 3), indicating only a small percentage of the organisms in the basal ice that are entering the lake and surviving there. When we studied this area previously by fluorescence and transmission electron microscopy, most of the cells were dead and distorted [13,14], indicating that they may have experienced a number of challenging stresses as the glacier rubbed against the bedrock, the organisms entered the lake water, and then were frozen into the accretion ice on the bottom of the glacier. Additionally, the concentrations of ions in the basal and accretion ice differ, creating yet another challenge for organisms in this region [9]. Proteobacteria and Actinobacteria were the predominant phyla in this ice (Figure 2). A few eukaryotes were present, and two archaea were evident. The few species that were in common with those in the basal ice included Betaproteobacteria, Actinobacteria, Gammaproteobacteria, and Cyanobacteria one arthropod, and one chlorophyte (Figure 2; Tables S3 and S8). Almost all of the organisms were most similar to soil, sediment, and aquatic species (Figure 4; Table S7), but there were higher numbers of halophilic and halotolerant species than in the basal ice, and very few organisms were psychrophilic, thermophilic, alkaliphilic, or acidophilic (Figure 5). Combined with our previous results that indicated severe damage in cells from this area, the conditions in this region appear to be more biologically challenging than in most other parts of the lake.

### 3.5. Middle Section of Embayment (3563 + 3585 m Sample – Type 1 and 2 Accretion Ice)

The 3563 + 3585 m sample (reanalyzed from data in [1,2]) from the middle of the shallow embayment was very different from all of the other samples in several respects. It produced the highest number of unique sequences (1214 at ≥97% identity; Figure 2). Of the 1056 bacteria, Firmicutes and Proteobacteria were in the highest numbers, with smaller, but similar, numbers of Cyanobacteria, Actinobacteria, and Bacteroidetes. The eukaryotes were primarily from the Fungi and Archaeplastida (probably pollen, which likely entered as trapped cells in the meteoric ice). The organisms indicated by the sequences are higher in almost all categories than for any other ice core sample. This includes soil/sediment, aquatic, marine, psychrophilic/psychrotolerant, thermophilic/thermotolerant, alkaliphilic/alkalitolerant, acidophilic/acidotolerant, desiccation-resistant, animal-associated, and plant-associated (Figure 4 and Figure 5; Tables S4, S7 and S8). The only exceptions are that there are more ice-associated organisms in the basal ice sample (3501 + 3520 m), and more halophilic/halotolerant organisms indicated in the eastern-most shallow embayment sample (3585 m; Table S5). This is consistent with more ice melting and ablation occurring in the basal ice for the ice-associated organisms; and is consistent with the ion concentrations reported previously [9], which showed a gradual increase in the ion concentrations, especially Na^+^ (which increased from 4 µmol/L at 3540 m, to >40 µmol/L at 3575 m, then decreased to 5 µmol/L at 3585 m), Mg^2+^ (which increased from 8 µmol/L at 3570 m to 30 µmol/L at 3572 m, and decreased to 3 µmol/L by 3580 m), SO_4_^2−^ (which slowly increased from 1 µmol/L from 3540 m to 3572 m, with a rapid increase to 32 µmol/L at 3572 m, followed by a rapid decrease to 1 µmol/L by 3585 m), and Cl^−^ (which gradually increased from 2 µmol/L at 3540 m to 30 µmol/L at 3572 m, followed by a decrease to 2 µmol/L by 3585 m). Thus, from the western section of the embayment to the eastern side of the embayment, levels of Na^+^, Mg^2+^, SO_4_^2−^, and Cl^−^ increased, peaking around the 3570 to 3575 m depths (Figure 1), followed by decreases by 3590 m. The concentration of Ca^2+^ remained between 1 and 5 µmol/L from 3540 m 3588 m, but then increased to 32 µmol/L by 3592 m, before decreasing to 1 µmol/L by 3605 m. The concentration of K^+^ remained at a constant concentration of 1 µmol/L throughout the accretion ice. 

In addition to ion concentration comparisons, total amino acid concentrations and non-purgable organic carbon (NPOC) concentrations also were compared. Each of these was lower in the accretion ice at 3540 m (approximately 10^−7^ and 10^−6^, mol/L respectively), and higher in the accretion ice between 3670 and 3605 m (approximately 3 × 10^−7^, and 2 × 10^−6^ mol/L), and decreased rapidly to approximately 10^−8^, and 9 × 10^−7^ moL/L (respectively) by 3620 m. There were positive associations between the numbers of species and concentrations of Na^+^ (*P* = 0.045), Ca^2+^ (*P* = 0.016), Mg^2+^ (*P* = 0.014), SO4^2−^ (*P* = 0.015), total amino acids (*P* = 0.004), and NPOC (*P* < 0.001). As these concentrations increased, so did the number of species that were observed.

The most striking characteristic of this sample was that it had high numbers of both thermophilic/thermotolerant and psychrophilic/psychrotolerant species. This suggests that the region between the 3563 and 3585 m accretion ice core sections (separated geographically be approximately 5 km, and temporally by approximately 2000 years; Figure 1), contains both hydrothermal and cold-water areas. The high numbers of alkaliphilic/alkalitolerant and acidophilic/acidotolerant species also are indicative of an influx of ions, such as are introduced by hydrothermal vents. As with the other ice core samples, most of the sequences in this region were unique to this sample, although some of the same species from this sample were present in other sections of the basal and accretion ice (Figure 3). Interestingly, several sequences of bacteria that were previously found in fish intestines were present in this sample [1,2] suggesting the presence of fish.

### 3.6. Eastern Section of Embayment (3,585 m Sample–Type 2 Accretion Ice)

For the eastern-most portion of the shallow embayment, represented by the 3585 m sample, the number of unique sequences was about 50% lower than in the sample from the middle of the embayment (3563 +3585 m; Figure 2). This is consistent with a decrease in concentrations of DNA, amino acids, NPOC and most ions, reported elsewhere [9]. However, while many types of organisms were lower in number in this sample, the numbers of organisms usually found in soil, sediment, aquatic, and marine environments remained high (Figure 4). Additionally, the numbers of thermophilic/thermotolerant and psychrophilic/psychrotolerant organisms also were moderately high, and the number of halophilic/halotolerant species was the highest of all samples and regions (Figure 5). This might indicate the influence of the hydrothermal activity, which is several kilometers away, or that an area of saltwater exists in this region, possibly as a brine layer. The concentration of Ca^2+^ peaked in this region, which might be the reason that higher numbers of halophilic and halotolerant species were found here. This region had primarily members of the Actinomyces and Proteobacteria, which is consistent with previous studies that have reported primarily organisms from the Actinomyces and Proteobacteria from adjacent ice core sections, and microscopical analyses that have reported fungi, bacteria (cocci and rod-shaped), diatoms, haptophytes, and plant pollen in several of the ice core sections from the shallow embayment [9,12,13,14,15,18,19,20]. As with the adjacent sample (middle section of the embayment, 3563 + 3585), molecular signals of the possibility of fish were found in this sample, including a sequence most similar to that from the fish pathogen, *Saprolignia parasitica*, and a sequence closest to *Notothenia coriiceps* (rockcod), a ray-finned fish common in the Southern Ocean near the shores of Antarctica, that has antifreeze proteins and lives at water temperatures between −1 and 4 °C.

### 3.7. Western Main Basin (3606 + 3621 m Sample–Type 1/2 Accretion Ice)

The number of unique sequences declined further in the accretion ice sample representing the western portion of the southern main basin (3606 +3621 m; reanalyzed from data in [1,2]). Only 129 unique sequences were found (93 at ≥97% identity), of which 105 were from Bacteria. More than 60% of those were from members of the Proteobacteria (Figure 2; Table S6). The remainder were split between the Firmicutes, Actinobacteria, and a few other phyla. Of the 24 eukaryotic sequences, almost all were fungi. The highest proportions of organisms indicated were psychrophilic/psychrotolerant, but absolute numbers were low (Figure 5). Additionally, there was a mixture of soil, sediment, aquatic, marine, animal-associated, and plant-associated organisms (Figure 4).

### 3.8. Habitats in Lake Vostok

Among the sequences found in all samples, more than one-third of the putative species were either psychrophilic or psychrotolerant (Figure 5). Also found were sequences from a number of halophilic and halotolerant species. These gradually increased from west to east in the embayment, with the highest numbers in the eastern-most section, which was approximately 9–10 km from the western shore (see Figure 1). The highest numbers of thermophilic, thermotolerant, psychrophilic, psychrotolerant species were found within the eastern half of the embayment, approximately 7–8 km from the western shore. Smaller, but substantial, numbers of sequences from desiccation-resistant, mesophiles, acidophiles, acidotolerant, alkaliphiles, and alkalitolerant species also were present in the same region (Figure 5; Table S7). 

The relative numbers of psychrophiles and psychrotolerant species is high in the basal ice, which was expected, but these drop in the western section of the shallow embayment. The declines in most categories of organisms might be due to the extreme conditions that probably exist there. This includes the scraping of the glacier and embedded rocks on the lake-bed, local thawing and freezing, and a transition from basal ice meltwater to lake water, each of which exhibit differences in ion concentrations [9]. There is a consistent increase in the number of halophilic and halotolerant taxa from the western section of the embayment to the extreme eastern portion. This is consistent with ion measurements, including Na^+^, K^+^, Mg^2+^, Ca^2+^, Cl^−^, and SO_4_^2−^, in the same regions [9]. However, in the extreme eastern portion of the embayment (3580 to 3600 m), where most of the ion concentrations decreased, Ca^2+^ concentrations increased greatly. Ice core section 3585 m (eastern embayment), which was within a region where Na+ concentrations were roughly half that of the peak values in the middle section of the embayment (approximately 3570 m), and where Ca^2+^ concentrations were low, had the highest number of halophilic/halotolerant species. These organisms may have been best suited to moderate Na^+^ concentrations.

### 3.9. Hydrothermal Activity

The large numbers of sequences from thermophiles and thermotolerant peaked in the eastern half of the embayment (Figure 5). This points to a possible hydrothermal source in the eastern portion of the embayment (Figure 1), which is consistent with previous reports of accretion ice from this region [1,2,10]. The middle region of the embayment (3563 + 3585 m) had the highest numbers of thermophilic/thermotolerant taxa, but also had the highest numbers of alkaliphilic/alkalitolerant, acidophilic/acidotolerant, and desiccation-resistant species, also suggesting hydrothermal activity within that region. However, the same sample also had the highest numbers of psychrophilic/psychrotolerant species. Because this sample is composed of two ice core sections that are approximately 3 km and 2000 years apart (see Figure 1), this apparent paradox might indicate a point source of hydrothermal activity that warms a narrow region of the embayment, which is surrounded by cold water. If a hydrothermal vent is present, it probably produces turbulence and mixing. The numbers of thermophilic/thermotolerant, psychrophilic/psychrotolerant, alkaliphilic/alkalitolerant, acidophilic/acidotolerant, and desiccation-resistant organisms decreased in the eastern region of the embayment (3585 m), and the same is true for the western section of the embayment (3540 + 3569 m; Figure 5). Therefore, higher numbers of total organisms, as well as thermophilic and thermotolerant organisms were suggestive of hydrothermal activity approximately between the 3570 and 3580 m ice core depths, which corresponds to a region approximately 7 km from the western shoreline (3 km from the peninsula to the east). This also coincides with the highest concentrations of ions, amino acids, LPOC, and DNA-containing cells [9]. 

### 3.10. Beyond the Peninsula

The peninsula that separates the embayment from the main basin is approximately 10 km from the western shore (Figure 1). The ice that accreted over the southern main basin to the east of the peninsula (the 3606 + 3621 m sample) has lower concentrations of sequences, including lower numbers of organisms and lower concentrations of ions [1,2,9,12,13,14] (Figure 2, Figure 3, Figure 4 and Figure 5). Reports further out in this region indicate that the levels of organisms and organic carbon continue at very low levels across the southern basin [10]. Therefore, the shallow embayment appears to be the area with the highest levels of biological activity, and relatively high sequence diversity (Shannon–Weaver index = 4.59, evenness = 0.44), possibly due to energy and chemical inputs from hydrothermal activity, as well as some chemical contributions from the basal ice. Conversely, the southern main basin appears to be extremely oligotrophic.

### 3.11. Potential Metabolic Capabilities

The BLAST results indicated that the vast majority of organisms in all samples were closest to heterotrophic species (Table 1; Tables S2–S9). The middle region of the embayment (3563 + 3585 m) had the highest number of heterotrophs, as well as the largest number of autotrophs (primarily chemolithoautotrophs). A number of forms of carbon fixation types were indicated, including the reductive pentose phosphate cycle (rPP cycle; Calvin–Benson cycle), reductive tricarboxylic acid (rTCA) cycle, reductive acetyl-CoA pathway, and the 3-hydroxypropionate pathway, possibly using chemosynthetic reactions and thermal energy from the hydrothermal vent to power these pathways. The first two carbon fixation pathways were the most prevalent. Organisms involved in carbon fixation, carbon cycling, C-1 metabolism, and hydrocarbon metabolic pathways were indicated in all of the samples, although the numbers were lowest in the basal ice and the western-most embayment sample (3540 + 3569 m sample). 

All of the steps in nitrogen cycling were represented in the sequences of organisms and genes found in the basal ice and in the accretion ice from the shallow embayment (Table 1; Tables S7–S9). This includes many species capable of nitrogen fixation, as well as those that perform nitrification, denitrification, ammonification, nitrogen reduction, assimilation, and decomposition. In addition, a few species that were indicated from the accretion ice in the middle and eastern portions of the shallow embayment that may be capable of anammox functions (Planctomycetes). Examples of all of these processes are far fewer in the sequences of organisms and genes in the western-most section of the shallow embayment, and in the main basin accretion ice, and there was no evidence for an anammox pathway in either location. In addition to these major functions, there were organisms and genes indicated among the sequences for iron oxidation, iron reduction, arsenite oxidation, arsenate reduction, and sulfate reduction present in both the basal and shallow embayment accretion ice (Table 1). Sequences of organisms and genes responsible for sulfur oxidation and reduction were found in the accretion ice from the embayment and the main basin. Organisms and genes for manganese oxidation and uranium oxidation were limited to the embayment accretion ice and basal ice, respectively.

The communities of organisms indicated from the sequences in the basal and accretion ice suggests representation in vital metabolic pathways. However, different sets of organisms were indicated in the ice core sections for analogous processes. For example, members of the Actinobacteria predominate in the basal ice, and may be responsible for nitrogen fixation (e.g., *Mycobacterium* spp. and *Streptomyces* spp.), nitrification (*Mycobacterium* spp. and *Streptomyces* spp.), denitrification (especially the Streptomycetes), assimilation (most organisms), and decomposition (Ascomycetes, Basidiomycetes, and others). Proteobacteria are potentially responsible for the other portions of nitrogen cycling (e.g., *Azoarcus* sp., *Nitrotoga* sp., *Nitrosococcus* sp., *Nitrosomonas* sp., and *Pseudomonas* spp.), although a few members of the Firmicutes (e.g., members of Clostridiales) may contribute to this process. While the total number of Actinobacteria is higher in the accretion ice samples compared to the basal ice sample, the numbers and proportions of members of the Proteobacteria and Bacteroidetes active in the nitrogen cycle predominate in the accretion ice samples (e.g., *Acidovorax* sp., *Azospirillum* sp., *Bacillus* spp., *Burkholderia* spp., *Denitrobacter* sp., *Diphorobacter* sp., *Mesorhizobium* sp., *Paracoccus* spp., and *Pseudomonas* spp.). However, there are some Actinobacteria potentially active in the nitrogen cycle in the accretion ice samples, including *Frankia* spp., *Micromonospora* spp., *Nocardia* sp., and *Streptomyces* spp.

Autotrophs indicated by the sequences are also varied within the ice core samples, with several types of carbon fixation present. Many were chemolithoautotrophs that utilize either hydrogen, sulfur, nitrogen, or iron to fuel the reactions. Many were found that were likely to be using the rPP cycle. Most obvious among this group were the Cyanobacteria, found in low abundance in several of the ice core samples, including the glacial ice, basal ice, and some of the accretion ice samples. Because no light reaches any of these regions, if the cyanobacterial cells are alive, they are probably functioning as heterotrophs. Viable cyanobacteria have been found previously in deep permafrost, which are capable of long-term survival in darkness [17]. Members of the Acidobacteria, Chlorobi, Alphaproteobacteria, Betaproteobacteria, and Gammaproteobacteria, whose sequences were found in the basal ice sample also use the rPP cycle to fix carbon dioxide into organic molecules. Within the accretion ice samples, a mostly different set of species within the Alphaproteobacteria, Betaproteobacteria, and Gammaproteobacteria are potentially capable of fixing carbon using the same pathway. 

The rTCA cycle was also represented, as indicated in the sequence data from the basal and accretion ice. Members of Chlorobi were present in both regions, but the percent identities were below the 97% level, and therefore, the presence of the rTCA cycle is tentative, at least for the basal ice sample. There were members of the Alphaproteobacteria, Deltaproteobacteria, and Epsilonproteobacteria in some of the accretion ice samples that suggested the presence of the rTCA cycle in the shallow embayment of Lake Vostok. Representation of the reductive acetyl-CoA pathway was indicated from the few members of the archaea, but this was not confirmed by any other sequence data. The 3-hydroxypropionate bicycle is present in some members of the Chlorobi and Alphaproteobacteria, but it was not possible to determine whether those found in the ice core sections used this pathway.

Metabolic systems dealing with iron and sulfur were common among the species indicated by sequences from the basal and accretion ice samples. This was expected because of the nearly universal importance of compounds containing these elements within most organisms. These reactions were found within members of the Acidobacteria, Actinobacteria, and Proteobacteria. Bacteria capable of C-1 metabolic processes (members of the Alphaproteobacteria and Betaproteobacteria), as well as those that have pathways for chromium and uranium metabolism (e.g., *Acidiphilium cryptum*, *Bacillus* spp.), were found only in the basal ice sample. This might indicate the presence of these components in the bedrock around the lake. Conversely, microbes that have metabolic capabilities for manganese compounds (e.g., *Caldimonas manganoxidans, Massilia pudita*) were found only in the accretion ice. Manganese compounds often are found in areas of hydrothermal activity, which is another indication that hydrothermal activity might exists within the shallow embayment.

### 3.12. Conceptual View of Lake Vostok

Based on the sum of the limnological studies from the Vostok 5G accretion ice core section, Lake Vostok appears to contain a complex ecosystem, which is concentrated near the suspected hydrothermal activity in the eastern region of the shallow embayment. The conditions and organisms in the embayment differ greatly from those found in the region of basal ice. The basal ice sequence data indicates that there exists a community of at least 513 distinct species (Figure 2; Table S2), of which at least 407 are bacteria (primarily Bacteroidetes and Proteobacteria), and at least 103 are eukaryotes (consisting of a mixture of members of the Alveolata, Animalia, Excavata, Fungi, Haptophyta, Heterokonta, and others). Among these are species that can carry out almost all steps in nitrogen cycling, including nitrogen fixation, nitrification, denitrification, nitrate reduction, assimilation, and decomposition (Table 1; Tables S1–S8). Additionally, while most of the species found were heterotrophic, there were many autotrophic species found as well, primarily chemolithoautotrophs, utilizing either the rPP or the rTCA cycles. Therefore, a community of organisms might be present that comprises a functioning ecosystem at the base of the glacier. Given the ions and nutrients present in the glacial ice and the bedrock, the ecosystem would be expected to be perpetual during the lifetime of the glacier. While the basal ice delivers some species to the lake water that survive there, most of the organisms in the basal ice are absent from the accretion ice (Figure 3), an indication of minimal input of viable organisms into the lake ecosystem from the glacial ice. While the number of viable organisms entering the lake from the basal ice is small, the overall influx of organic materials might be much larger. Within the shallow embayment, only limited mixing of the water from one region to another was apparent based on the sequence data (Figure 2 and Figure 3). This is also supported by other reports [10,12,13,14,15,18,19].

**Figure 6:**
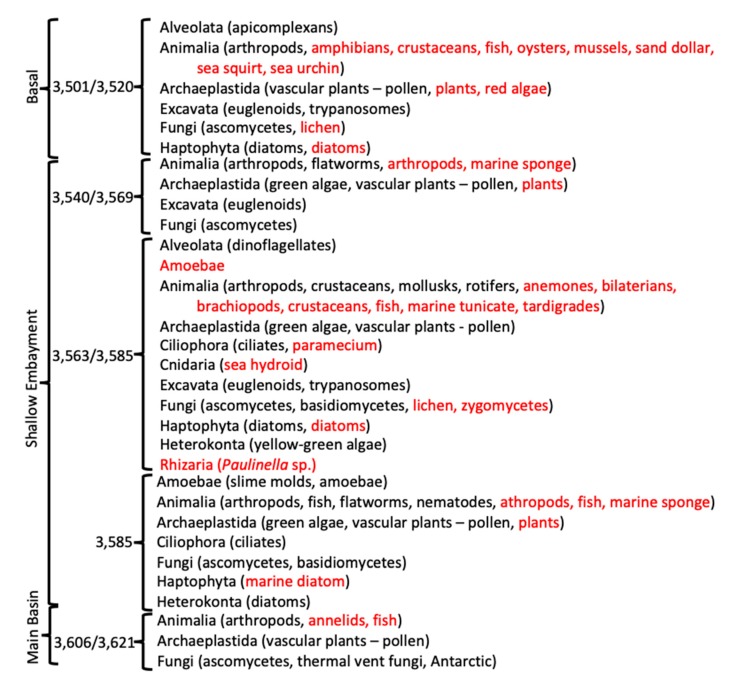
Condensed list of taxonomic groups of eukarya, based on sequence results. Black font indicates direct sequence BLAST matches to sequences from the NCBI database. Red font indicates eukaryotic taxa that have been reported to be associated with bacteria or other eukaryotic organisms.

The community of organisms within the embayment of Lake Vostok forms a more complex and diverse ecosystem that than the one indicated in the basal ice (Figure 1, Figure 2, Figure 3, Figure 4, Figure 5 and Figure 6). The western side of the embayment contains a restricted number and diversity of organisms, possibly due to several influences, including the entry of the glacier into the embayment, breaking of some of the pieces of basal ice, melting, friction, delivery of glacial flour to the region, turbulence, temperature gradients, ion gradients and freezing to the accretion ice layers. This appears to be a challenging environment for most organisms, as indicated by the low numbers of sequences, as well as previously reported low numbers of cells, viable cells, and isolates [9,12,13,14]. In the middle region of the ambayment, there are higher numbers of total cells, viable cells, number of sequences, and number of unique sequences [1,2,10,12,13,14,15,18,19,21]. This suggests the influence of the proposed hydrothermal activity, which might be adding energy and additional nutrients to the lake [1,2,9,22].

In addition to a wide range of bacterial species, many eukaryotic sequences were present, as well as some bacterial species that are normally associated with eukaryotes (Figure 6; Tables S2–S7). The basal ice contained sequences most similar to those of several aquatic arthropods, apicomplexans, diatoms, haptophytes, euglenoids, trypanosomes, plants, and ascomycetes. Cells of some of these, specifically diatoms, haptophytes, plant pollen, and fungi, have been observed microscopically from some of the Vostok ice cores [12,13,14]. Some of the bacterial species indicated from the sequence data are often associated with amphibians, crustaceans, fish, and plants. Additionally, sequence directly indicating species of arthropods, crustaceans, fish, mollusks, rotifers, algae, dinoflagellates, ciliates, euglenoids, trypanosomes, fungi, amoebae, nematodes, flatworms, and diatoms were found. These might simply be pieces of organisms that were deposited on, entrapped within, and released from the glacial ice. However, we found little evidence that substantial numbers of sequences in the accretion ice were derived from the meteoric glacial ice. 

The accretion ice had more unique sequences from eukaryotic species than did the basal ice, which suggested a more complex ecosystem in the lake (Figure 6). This included a number of small animals (e.g., arthropods, flatworms, crustaceans, mollusks, rotifers, fish, and nematodes). Additionally, a number of animals are suggested, because of the bacteria and protists that are normally associated with them (e.g., annelids, fish, marine sponges, anemones, bilaterians, brachiopods, and tardigrades). Sequences from plants are likely from pollen that is delivered to the lake water by the glacier. Pollen has been reported from microscopic examination of the accretion ice meltwater (e.g., [20]). Sequences from green algae were detected throughout the shallow embayment, while they were absent from the basal ice sample, indicating that they might have originated in the shallow embayment. While they are not photosynthetically active, they may be able to survive heterotrophically, as do some algae and cyanobacteria. Ascomycetous fungi were found in all of the ice core sections, while basidiomycete sequences were found only in accretion ice from the middle and eastern portions of the shallow embayment, and one possible zygomycete species was found in the middle section of the shallow embayment. Many of the same ascomycete and basidiomycete species were isolated in our previous studies of the Vostok accretion ice (and glacial) cores [13,14]. There were a few species of amoebae and slime molds indicated in the accretion ice from the shallow embayment, as well as many species of protists, including ciliates, diatoms, dinoflagellates, yellow-green algae, euglenoids, trypanosomes, heterokonts, and rhizarians. Therefore, Lake Vostok may contain a functioning ecosystem that receives chemical and energy inputs from the overriding glacier and from possible hydrothermal sources.

## Figures and Tables

**Table 1:** Potential metabolic capabilities for the organisms present in basal and accretion ice, based on sequence results from this research, in addition to results reported in refs. [1,2]. Details of the species are in Tables S2–S9. Abbreviations: Ac—Actinobacteria; αP—Alphaproteobacteria; Ar—Archaea; As—Ascomycota; Ba—Bacteroidetes; Bd—Basidiomycota; βP—Betaproteobacteria; Cb—Chlorobi; Ch—Chlorophyta; Cy—Cyanobacteria; DT—Deinococcus-Thermus; δP—Deltaproteobacteria; εP—Epsilonproteobacteria; Fi—Firmicutes; γP—Gammaproteobacteria; Pl—Planctomycetes.

Process	Basal Ice	Lake Vostok Accretion Ice
Nitrogen fixation (N_2_→ NH_4_^+^)	Ac, Fi, αP, βP	Ac, Cy, Fi, αP, βP, εP,γP
Nitrification (NH_4_^+^→NO_2_^−^)	βP	αP, βP
Nitrification (NO_2_^−^→NO_3_^−^)	Ac	αP, βP
Denitrification (NO_2_^−^→ NH_4_^+^)	Ac, DT, αP, βP, δP,γP	Fi, αP, βP, γP
Denitrification (NO_3_^−^→NO_2_^−^)	Ac	Fi, αP, βP, γP
Denitrification (NO_3_^−^→N_2_)	Ac, DT, αP, βP, δP,γP	Fi, αP, βP, γP
Nitrate reduction (NO_3_^−^→ NH_4_^+^)	δP,γP	Fi, αP, βP, γP
Assimilation (NH_4_^+^→organic)	many	many
Decomposition (organic→NH_4_^+^)	Ac, γP, As, Bd, other heterotrophs	As, Bd, other heterotrophs
Anammox (NH_4_^+^+NO_2_^−^→N2)	Pl	Pl
Carbon fixation Reductive PP Reductive TCA Reductive acetyl-CoA 3-hydroxypropionate bicycle	Ad, Ch, Cy, αP, βP,γP Cb Ar αP	Cy, αP, βP, γP Cb, αP, δP,εP Ar Cb
C-1 metabolism	αP, βP	αP, βP
Arsenic oxidation	αP	βP
Arsenate reduction	αP	βP
Chromium reducing	Ac, αP, βP	nd
Iron oxidation	βP	βP
Iron reduction	αP, βP, δP	δP
Manganese oxidation	nd	δP
Methylotrophs	αP, βP	βP, As
Sulfur oxidation	βP, γP	βP
Sulfur (sulfate) reduction	βP, δP	δP
Uranium oxidation	Fi, βP	nd
Uranium reduction	Fi, δP	nd

## Supplementary Materials

The following are available online at https://www.mdpi.com/2079-7737/9/3/55/s1, Supplemental File S1: Python scripts.; Table S1: Characteristiccs of ogranisms in basal ice.; Table S2: Characteristics of organisms in the western region of embayment.; Table S3: Characteristics of organisms in the middle portion of the embayment.; Table S4: of organisms in the eastern portion of the embayment; Table S5: Characteristics of organisms in the western main basin of Lake Vostok; Table S6: Species and isolates listed by characteristics; Table S7: Enumeration of sequence reads and unique sequences; Table S8: Number of reads per gene.

## References

[1] Rogers S.O., Shtarkman Y.M., Koçer Z.A., Edgar R., Veerapneni R.S., D’Elia T., Morris P.F. (2013). Subglacial Lake Vostok (Antarctica) Accretion Ice Contains a Diverse Set of Sequences from Aquatic, Marine and Sediment-Inhabiting Bacteria and Eukarya. Biology.

[2] Shtarkman Y., Koçer Z., Edgar R., Veerapaneni R., D’Elia T., Morris P., Rogers S.O. (2013). Subglacial Lake Vostok (Antarctica) Accretion Ice Contains a Diverse Set of Sequences from Aquatic, Marine and Sediment-Inhabiting Bacteria and Eukarya. PLoS ONE.

[3] Wright A., Siegert M. (2012). A Fourth Inventory of Antarctic Subglacial Lakes. Antarct. Sci..

[4] Denton G.H., Sugden D.E., Marchant D.R., Hall B.L., Wilch T.I. (1993). East Antarctic Ice Sheet Sensitivity to Pliocene Climatic Change from a Dry Valleys Perspective. Geogr. Ann. Ser. A Phys. Geogr..

[5] Petit J.R., Jouzel J., Raynaud D., Barkov N.I., Barnola J.M., Basile I., Benders M., Chappellaz J., Davis M., Delaygue G. (1999). Climate and Atmospheric History of the Past 420,000 Years from the Vostok Ice Core, Antarctica. Nature.

[6] Siegert M.J., Ridley J.K., Kapitsa A.P., de Q Robin G., Zotikov I.A. (1996). A Large Deep Freshwater Lake Beneath the Ice of Central East Antarctica. Nature.

[7] Ferraccioli F., Finn C.A., Jordan T.A., Bell R.E., Anderson L.M., Damaske D. (2011). East Antarctica rifting triggers uplift of the Gambutsev Mountains. Nature.

[8] Siegert M.J., Ellis-Evans J.C., Tranter M., Mayer C., Petit J.R., Salamatin A., Priscu J.C. (2001). Physical, Chemical and Biological Processes in Lake Vostok and Other Antarctic Subglacial Lakes. Nature.

[9] Christner B., Royston-Bishop G., Foreman C., Arnold B., Tranter M., Welch K., Lyons B., Tsapin A., Studinger M., Priscu J. (2006). Limnological Conditions in Subglacial Lake Vostok, Antarctica. Limnol. Oceanogr..

[10] Bulat S.A., Alekhina I.A., Lipenkov V.Y., Lukin V.V., Marie D., Petit J.R. (2009). Cell Concentrations of Microorganisms in Glacial and Lake Ice of the Vostok Ice Core, East Antarctica. Microbiology.

[11] Salamatin A.N., Tsyganova E.A., Lipenkov V.Y., Petit J.R. (2004). Vostok (Antarctica) Ice-Core Time-Scale from Datings of Different Origins. Ann. Glaciol..

[12] Abyzov S.S., Poglazova M.N., Mitskevich J.N., Ivanov M.V., Castello J.D., Rogers S.O. (2005). Common features of microorganisms in ancient layers of the Antarctic ice sheet. Life in Ancient Ice.

[13] D’Elia T., Veerapaneni R., Rogers S.O. (2008). Isolation of Microbes From Lake Vostok Accretion Ice. Appl. Environ. Microbiol..

[14] D’Elia T., Veerapaneni R., Theraisnathan V., Rogers S.O. (2009). Isolation of Fungi from Lake Vostok Accretion Ice. Mycologia.

[15] Karl D., Bird D.F., Björkman K., Houlihan T., Shackelford R., Tupas L. (1999). Microorganisms in the Accreted Ice of Lake Vostok, Antarctica. Science.

[16] Rogers S.O., Bendich A.J. (1985). Extraction of DNA from milligram amounts of fresh, herbarium and mummified plant tissues. Plant Mol. Biol..

[17] Vishinetskaya T.A., Erokhina L.G., Spirina E.L., Shatilovich A.V., Vorobyova E.A., Tsapin A.I., Gilichinsky D.A., Castello J.D., Rogers S.O. (2005). Viable phototrophs: Cyanobacteria and green algae from the permafrost darkness. Life in Ancient Ice.

[18] Bell R.E., Studinger M., Tikku A.A., Clarke G.K., Gutner M.M., Meertens C. (2002). Origin and Fate of Lake Vostok Water Frozen to the Base of the East Antarctic Ice Sheet. Nature.

[19] Bell R., Studinger M., Tikku A., Castello J.D., Castello J.D., Rogers S.O. (2005). Comparative Biological Analyses Of Accretion Ice from Subglacial Lake Vostok. Life in Ancient Ice.

[20] Sambrotto R., Burckle L., Castello J.D., Rogers S.O. (2005). The nature and likely sources of biogenic particles found in ancient ice from Antarctica. Life in Ancient Ice.

[21] Priscu J.C., Adams E.E., Lyons W., Voytek M.A., Mogk D.W., Brown R.L., McKay C.P., Takacs C.D., Welch K.A., Wolf C.F. (1999). Geomicrobiology of Subglacial Ice Above Lake Vostok, Antarctica. Science.

[22] Bulat S.A., Alekhina I.A., Blot M., Petit J.R., De Angelis M., Wagenbach D., Lipenkov V.Y., Vasilyeva L.P., Wloch D.M., Raynaud D. (2004). DNA Signature of Thermophilic Bacteria from the Aged Accretion Ice of Lake Vostok, Antarctica: Implications for Searching for Life in Extreme Icy Environments. Int. J. Astrobiol..

