# Supplementary material for: Metatranscriptomic and Metagenomic Analysis of Biological Diversity in Subglacial Lake Vostok (Antarctica)
Source: Biology (Basel). 2020 Mar 16;9(3):55. doi: 10.3390/biology9030055 (PMC7150893; doi:10.3390/biology9030055)
Supplement: Supplementary file 1 [file biology-09-00055-s001.pdf]

Supplementary File S1. Python scripts.

• This script will demultiplex the raw FASTQ sequence files and remove the MID sequence from the sequences. A new file is then created corresponding to each of the samples.

```
file1 = #input file
file2 = #MID2 – 2149m sample
file3 = #MID4 – 3501m + 3520m samples
file4 = #MID5 – 3540m + 3569m samples
file5 = #MID6 – 3585m
file6 = #MID10 – negative control

# creates empty lists for each sample
mid2 = []
mid4 = []
mid5 = []
mid6 = []
mid10 = []

# MID indexing sequences
primermid2 = ['ACGCTCGACA', 'TGTCGAGCGT']
primermid4 = ['AGCACTGTAG', 'CTACAGTGCT']
primermid5 = ['ATCAGACACG', 'CGTGTCTGAT']
primermid6 = ['ATATCGCGAG', 'CTCGCGATAT']
primerneg = ['TCTCTATGCG', 'CGCATAGAGA']

import Bio
from Bio import SeqIO
```

```
for seq_record in SeqIO.parse(file1, 'fastq'):

    if 'ACGCTCGACA' in seq_record.seq[-10:] or 'TGTCGAGCGT' in seq_record.seq[-10:]: # MID2 – 2149
        trimmed = (rec[-10:] for rec in \
                 SeqIO.parse(file1, "fastq") \
                 if rec.seq.startswith("ACGCTCGACA") \
               or rec.seq.startswith('TGTCGAGCGT'))
        count = SeqIO.write(trimmed, file2, "fastq")
        print("Saved %i reads" % count)
        print('2149 done')

    if 'AGCACTGTAG' in seq_record.seq[-10:] or 'CTACAGTGCT' in seq_record.seq[-10:]: # MID4 – 3501 + 3520
        trimmed = (rec[-10:] for rec in \
                 SeqIO.parse(file1, "fastq") \
                 if rec.seq.startswith("AGCACTGTAG") \
               or rec.seq.startswith('CTACAGTGCT'))
        count = SeqIO.write(trimmed, file3, "fastq")
        print("Saved %i reads" % count)
        print('3501+3520 done')

    if 'ATCAGACACG' in seq_record.seq[-10:] or 'CGTGTCTGAT' in seq_record.seq[-10:]: # MID5 – 3540 + 3569
        trimmed = (rec[-10:] for rec in \
                 SeqIO.parse(file1, "fastq") \
                 if rec.seq.startswith("AGCACTGTAG") \
               or rec.seq.startswith('CTACAGTGCT'))
        count = SeqIO.write(trimmed, file4, "fastq")
        print("Saved %i reads" % count)
        print('3540+3569 done')

    if 'ATATCGCGAG' in seq_record.seq[-10:] or 'CTCGCGATAT' in seq_record.seq[-10:]: # MID6 – 3585
        trimmed = (rec[-10:] for rec in \
                 SeqIO.parse(file1, "fastq") \
```

```
            if rec.seq.startswith("ATATCGCGAG") \
          or rec.seq.startswith('CGTGTCTGAT'))
    count = SeqIO.write(trimmed, file5, "fastq")
    print("Saved %i reads" % count)
    print('3585 done')

if 'TCTCTATGCG' in seq_record.seq[-10:] or 'CGCATAGAGA' in seq_record.seq[-10:]: # MID10 – neg control
    trimmed = (rec[-10:] for rec in \
            SeqIO.parse(file1, "fastq") \
            if rec.seq.startswith("TCTCTATGCG") \
          or rec.seq.startswith('CGCATAGAGA'))
    count = SeqIO.write(trimmed, file6, "fastq")

    print("Saved %i reads" % count)
    print('neg control done')
```

• The goal of this program is to remove duplicate GI numbers from each input file. For each GI number, the sample with the highest % identity is assumed to be the best. If the percent identity is tied for multiple samples, then the lowest e-value is the chosen sample among the tied percent identity.

```
# file to have duplicates removed
file1 = # input file
f1 = open(file1,'r')

# output file
file2 = # output file
f2 = open(file2,'w')
f1.readline() # bypasses header in file
best_samp = {}

# creates dictionary for each gi number found first.
for read in f1:
    gi = read.split(',')[2]
    if gi not in best_samp:
        best_samp[gi] = read

# resets cursor to top of file and skips header
f1.seek(0)
f1.readline()

# begins 2nd read through and replaces item if better
for read2 in f1:
    gi2 = read2.split(',')[2]
```

```
    pid_new = read2.split(',')[6]
    pid_new = float(pid_new)

    pid_org = best_samp[gi2].split(',')[6]
    pid_org = float(pid_org)

    if pid_new > pid_org:
            best_samp[gi2] = read2
    if pid_new < pid_org:
        pass
    if pid_new == pid_org:
        e_val_new = read2.split(',')[7]
        e_val_old = best_samp[gi2].split(',')[7]
        if e_val_new < e_val_old:
            best_samp[gi2] = read2

for key,value in best_samp.items():
    clean_val = str(best_samp[key])
    clean_val = clean_val.replace('\'','').replace('"','').replace('[','').replace(']','').replace('\n','')
    f2.write(clean_val + ',' + '\n')


f1.close()
f2.close()
```

• This script removes the EcoRI NotI primers from the reads.
Based upon the Biopython Cookbook script.

```
import Bio
from Bio import SeqIO

file = # input file
out = #output file

trimmer = (rec[19:] for rec in \
   SeqIO.parse(file,'fasta') \
   if rec.seq.startswith('AATTCGCGGCCGCGTCGAC'))

SeqIO.write(trimmer,out,'fasta')
```

- This script uses Biopython to parse a FASTA file. Then the built in Python statistics module is used to analyze the mean, median, mode, maximum, and minimum read lengths.

```
from Bio import SeqIO
import statistics

file1 = # input file
print(file1.split('\\')[-1])
records = list(SeqIO.parse(file1, "fasta"))
print("Total reads: %i" % len(records))

sizes = [len(rec) for rec in records]


print("Mean read length:", statistics.mean(sizes))
print("Median:", statistics.median(sizes))
print("Mode:", statistics.mode(sizes))
print("Max:", max(sizes))
print("Min:", min(sizes))
```

• This script uses Biopython to retrieve full GenBank files based upon accession numbers. The GenBank reports are then parsed to determine what the product is of the particular gene is.

```
from Bio import Entrez
from Bio import SeqIO
from Bio.SeqFeature import FeatureLocation

count = 1
products = []

file1 = # input file
f1 = open(file1, 'r')

file2 = # Output file
f2 = open(file2, 'w')
f1.readline()
for read in f1:
    temp_list = []
    accession = read.split(',')[1] #retrieve accession from file
    read = read.replace('\n','')
    temp_list.append(read)

# retrieve start and end locations
    hit_start = read.split(',')[4]
    hit_end = read.split(',')[5]
```

```
    start = str(hit_start)
    end = str(hit_end)

    start2 = start.replace('\'','').replace('"','').replace('[','').replace(']','')
    end2  = end.replace('\'','').replace('"','').replace('[','').replace(']','')

    start3 = float(start2)
    int_1_s = int(start2)

    end3 = float(end2)
    int_2_e = int(end2)

# confirms start is smaller than end or rearranges
    if int_1_s > int_2_e:
        int_start = int_2_e
        int_end = int_1_s
    else:
        int_start = int_1_s
        int_end = int_2_e

    hit_range = range(int_start, int_end)

    print(accession + ' being retrieved from NCBI...')

    Entrez.email = # user email

    #fetch full genbank report from NCBI
    handle = Entrez.efetch(db='nucleotide', id= accession, rettype='gb', retmode='text')
    record = SeqIO.read(handle,'genbank') #assign handle to variable for manipulation
    feature_count = len(record.features) # counts number of features on report

    for i in range(1, feature_count):
```

```
        feature = record.features
        f = feature[i].location
        range1 = list(f)
        range2 = list(hit_range)
        if any(elem in range2 for elem in range1):
            try:
                prod = feature[i].qualifiers['product']
                prod = str(prod)
                clean_prod = prod.replace('\'','').replace("'",'').replace('[','').replace(']','')
                temp_list.append(clean_prod)
                print(prod + ' found for ' + accession)
            except:
                pass
    products.append(temp_list)
    print(str(count) + ' retrieved so far...')
    count += 1

for i in products:
    clean_products = str(i)
    clean_products = clean_products.replace('\'','').replace("'",'').replace('[','').replace(']','')
    clean_products += '\n’\
    f2.write(clean_products)

f1.close()
f2.close()
```

• This script uses Biopython to retrieve the phylum of an organism using the taxonomic ID number retrieved during initial BLAST analysis.

```
from Bio import Entrez
Entrez.email = #email
file1 = #input file
file2 = #output file

def find(lst, key, value):
    for i, dic in enumerate(lst):
        if dic[key] == value:
            return i

master = [ ]

with open(file1,'r') as f1:
    header = f1.readline()
    header = header.split(',')[:13]
    header = str(header)
    header = header.replace('\n','').replace('\'','').replace('"','').replace('[','').replace(']','')
    master.append(header + ',' + 'phylum')

    for read in f1:
        temp = [ ]
        read2 = read.split(',')[:13]
```

```
        read2 = str(read2)
        read2 = read2.replace('\n','').replace('\'','').replace('"','').replace('[','').replace(']','')
        temp.append(read2)
        taxid = read.split(',')[8]
        handle = Entrez.efetch(db="Taxonomy", id=taxid, retmode="xml")
        records = Entrez.read(handle)
        taxa = records[0]["LineageEx"]
        taxa = list(taxa)
        index = find(taxa,'Rank','phylum')

        if index != None:
            phylum = taxa[index]
            sciname = phylum['ScientificName']
            sciname = sciname.replace(',','').replace('\'','').replace('"','').replace('[','').replace(']','')
            print(sciname)
            temp.append(sciname)
        else:
            temp.append('none')
        master.append(temp)

with open (file2,'w') as f2:
    for i in master:
        item = str(i)
        item = item.replace('\'','').replace('"','').replace('[','').replace(']','')
        f2.write(item + '\n')
```

Table S1. Characteristics of organisms in basal ice. Taxa in the basal ice sample (3,501 + 3,520 m sections), where characteristics could be determined.

Taxonomy	Species/Strain/Isolate	Characteristics (habitat, habit, functions)
**ARCHAEA**		
Euryarchaeota	*Halorubrum trapanicum*	Halophilic
**BACTERIA**		
Actinobacteria	*Actinomyces naeslundii*	animal associated
	Actinomyces oris	animal associated
	Actinomyces radicidentis	animal associated
	Actinoplanes sp. SE50/110	soil
	Cryobacterium sp. GCJ02	soil, psychrophilic
	Cryobacterium sp. LW097	soil, psychrophilic
	Dietzia timorensis	soil
	Euzebya sp. DY32-46	deep ocean
	Gordonia iterans	various environmental sources
	Hathewaya histolytica	psychrophilic
	Lysinimonas sp.	soil
	Microlunatus phosphovorus NM-1	sediment
	Mycobacterium sp. PYR15	estuary wetland, nitrogen fixation
	Nakamurella panacisegetis	soil
	Phycicoccus dokdonensis	soil
	Pseudolabrys taiwanensis	soil
	Rhodococcus jostii RHA1	soil
	Stenotrophomonas sp. G4	marine, nitrogen fixation

	Streptomyces hygroscopicus<br>*Streptomyces iranensis*<br>*Streptomyces parvulus*<br>*Streptomyces* sp. 11-1-2<br>*Streptomyces* sp. 3211<br>*Streptomyces* sp. Sge12 –<br>*Streptomyces* sp. WAC00288	soil<br>soil<br>soil<br>soil, nitrogen fixation, plant associated<br>soil<br>forest soil, nitrogen fixation<br>soil
Bacteroidetes	*Aequorivita sublithinicola*<br>*Alkalitalea saponilacus*<br>*Arenibacter algicola*<br>*Bacteroides caccae*<br>*Bacteroides caecimuris*<br>*Bacteroidetes bacterium*<br>*Bacteroides fragilis*<br>*Bacteroides uniformis*<br>*Chryseobacterium taklimakanense*<br>*Chryseobacterium* sp. 3008163<br>*Cyclobacterium amurskyense*<br>*Cyclobacterium marinum*<br>*Dyadobacter fermentans*<br>*Echinicola strongylocentroti*<br>*Echinicola vietnamensis*<br>*Elizabethkingia meningoseptica*<br>*Flavobacterium anhuiense*<br>*Flavobacterium arcticum*<br>*Flavobacterium branchiophilum*<br>*Flavobacterium branchiophilum* FL<br>*Flavobacterium columnare*<br>*Flavobacterium crassostreae*<br>*Flavobacterium gilvum*	Isolated from Antarctic quartz stone<br>alkaliphilic, soda lake<br>from marine diatom (*Skeletonema marinoi* ST54)<br>animal gut<br>animal gut<br>freshwater lake<br>animal gut<br>animal gut<br>permafrost, tundra soil, high Arctic soil<br>soil, plant associated<br>marine, heterotrophic<br>marine, from sand dollar, coastal marine wetland<br>soil, plant associated, nitrogen fixation<br>marine, from sea urchin (*Stronglyocentrotus intermedius*)<br>marine, sea water (mussel farm), halotolerant<br>water, marine, soil, from fish & amphibians<br>soil<br>Arctic marine<br>aquatic, fish pathogen (salmonid)<br>cold fresh water, fish pathogen<br>fish pathogen (catfish)<br>isolated from Pacific oyster<br>stream water<br>warm spring

	Flavobacterium indicum	soil, freshwater
	Flavobacterium johnsoniae	Antarctic penguin feces
	Flavobacterium kingsejongi	
	Flavobacterium psychrophilum	cold aquatic, fish pathogen (salmonid)
	Flavobacterium sp. 140616W15	cold freshwater, fish
	Flavobacterium sp. CJ74	saline Antarctica lake
	Flavobacterium sp. HYN0049	lake water
	Flavobacterium sp. MEBiC07310	tidal flat sediment
	Candidatus Fluviicola riflensis	aquifer
	Fluviicola taffensis	freshwater
	Gillisia sp. Hel1_33_143	cold marine
	Gramella sp. SH35	tidal flat
	Hymenobacter sedentarius	soil
	Hymenobacter sp. PAMC 26554	from Antarctic lichen
	Kordia sp. SMS9	associated with diatoms
	Maribacter sp.	marine
	Maribacter sp. 1_2014MBL_MicDiv	marine sediment, sea water
	Maribacter sp. T28	marine
	Marinifilaceae bacterium SPP2	Antarctic marine sediment
	Mariniflexile sp. TRM1-10	marine, associated with brown algae
	Marivirga tractuosa	beach sand
	Mucilaginibacter gotjawali	forest soil
	Mucilaginibacter sp. BJC16-A31	soil
	Mucilaginibacter sp. HYN0043	cold soil, permafrost
	Myroides odoratimimus	freshwater, opportunist (on animals)
	Myroides profundi	deep-sea sediment
	Pedobacter cryoconitis	psychrotolerant, from alpine glacier cryoconite
	Pedobacter heparinus	soil
	Pedobacter steynii	stream
	Prevotella jejuni	animal gut
	Pseudarcicella sp.	associated with annelids (leeches)

	Pseudopedobacter saltans<br>*Sediminicola* sp.<br>*Sediminicola* sp. YIK13<br>*Solitalea canadensis*<br>*Sphingobacterium mizutaii*<br>*Sphingobacterium* sp. 21<br>*Sphingobacterium* sp. B29<br>*Tamlana* sp. UJ94<br>*Weeksella virosa*	cold soil<br>marine sediment<br>marine<br>cold soil, freshwater<br>soil<br>Antarctic soil<br>soil, animal associated<br>marine<br>animal associated
Cyanobacteria	*Arthrospira* sp. PCC 8005	alkaline lakes
Elusimicrobia	*Elusimicrobium* sp.	protozoan endosymbiont
Firmicutes	*Bacillus cereus*<br>*Blautia coccoides*<br>*Caecibacter massiliensis*<br>*Clostridium pasteurianum*<br>*Dialister pneumosintes*<br>*Eubacterium siraeum*<br>*Lactobacillus plantarum*<br>*Megasphaera hexanoica*<br>*Negativicoccus massiliensis*<br>*Paenibacillus polymyxa* ATCC 842<br>*Paenibacillus* sp. FSL R7-0331<br>*Paenisporosarcina* sp.<br>*Staphylococcus* sp.	soil<br>animal gut<br>animal gut<br>nitrogen fixation<br>animal associated<br>animal associated<br>animal gut<br>animal gut<br>animal gut<br>soil<br>soil, water<br>psychrophilic, Antarctica<br>animal associated
Nitrospinae	uncultured *Nitrospina* sp.	marine, nitrite-oxidizing
Planctomyces	*Paludisphaera borealis*	northern peat bog and tundra

Proteobacteria		
α-proteobacteria	*Acidiphilium multivorum* AIU301	metabolism of arsenate/arsenite compounds, pyrite mine
	Acidiphilium cryptum JF-5	chromate (IV) reduction, iron (III) reduction
	Azorhizobium caulinodans ORS 571	nitrogen fixer, plant associated
	Pseudolabrys taiwanensis	soil
	Candidatus Filomicrobium marinum	cold marine, methylotroph
	Methylorubrum extorquens	methylotrophic, plant associated
β-proteobacteria	*Achromobacter ruhlandii*	soil
	Achromobacter xylosoxidans	moist environments
	Acidovorax carolinensis	soil
	Acidovorax cattleyae	soil, plant associated
	Acidovorax sp. 1608163	sediments
	Azoarcus sp.	nitrogen fixation
	Azoarcus sp. DN11	groundwater, denitrification
	Dechloromonas sp.	nitrite reduction, nitrogen fixation, assimilation
	Delftia acidovorans	soil, rock
	Ferribacterium sp.	freshwater lake sediment, iron (III) reduction
	Ferriphaselus amnicola	groundwater, iron-oxidizing
	Hydrogenophaga sp. RAC07	aquatic
	Kineococcus radiotolerans	radiation tolerant, halotolerant, aquatic, endolithic comm.
	Limnohabitans australis	freshwater
	Limnohabitans sp. MMS-10A-192	freshwater lakes
	Limnohabitans sp. Rim11	freshwater
	Limnohabitans sp. Rim49	freshwater (streams and lakes)
	Limnohabitans sp. SP2	planktonic freshwater lakes and streams
	Massilia buxea	rock surface on lake shore
	Massilia glaciei	glacier (Tibet)
	Massilia putida	isolated from wolfram (manganese tungstate) mine
	Methylophilus leisingeri	soil, methylophilic

	Methylophilus sp.	freshwater, methylotroph
	Candidatus Methylopumilus planktonicus	pelagic freshwater methylotroph, planktonic, lake
	Methylorubrum extorquens	soil, plant associated, methylotroph
	Methylotenera sp.	lake sediment methylotroph
	Methylotenera versatilis	lake sediment, methylotroph
	Methylovorus glucosotrophus	marine methylotroph
	Methylovorus glucosetrophus SIP3-4	lake methylotroph
	Methylovorus sp. MP688	soil methylotroph
	Candidatus Nitrotoga sp.	low temperature, nitrite oxidizer (nitrification)
	uncultured *Nitrosomonas* sp.	ammonia oxidation to nitrite (nitrification)
	Pandoraea oxalativorans	environmental, various
	Paraburkholderia bannensis	acidic swamp
	Paraburkholderia mimosarum	soil, plant associated
	Paraburkholderia phymatum STM815	soil, plant associated, nitrogen fixation
	Raoultella planticola	water, soil, fish
	Rhizobacter gummiphilus	soil
	uncultured *Rhodoferax* sp.	aquatic, psychrotolerant
	Sulfuriferula thiophila	hot spring, sulfur-oxidizing, chemolithoautotrophy
	Thauera aromatica K172	NO reduction
	Thauera hydrothermalis	thermophilic, hot spring
	Thauera sp. K11	river sediment
	Undibacterium amnicola	stream water
	Undibacterium sp.	freshwater stream
	Variovorax boronicumulans	soil
	Variovorax sp. PMC12	soil, plant associated
δ-proteobacteria	*Anaeromyxobacter* sp. Fw109-5	subsurface sediments, metal reducing
	uncultured *Desulfobacterium* sp.	sediment, water, sulfate reducing
	Desulfococcus multivorans	sediments, sulfate reducing, marine
	Desulfococcus oleovorans	mud
	Desulfurella acetivorans	thermophilic, sulfur-reducing, cyanobacterial mat

	Geobacter sp.<br>uncultured Geobacteraceae bacterium<br>*Hippea maritima*<br>*Syntrophus aciditrophicus*	organic and metal oxidation<br>iron (II) reduction, manganese reduction<br>marine hydrothermal vent sediments, sulfur-reducing<br>sediment
γ-proteobacteria	*Aeromonas hydrophila*<br>*Brenneria nigrifluens*<br>*Buchnera aphidicola*<br>*Cellvibrio gandavensis*<br>uncultured *Cellvibrio* sp.<br>*Enterobacter* sp.<br>*Gilvimarinus* sp.<br>uncultured *Nitrosococcus* sp.<br>*Klebsiella pneumoniae*<br>*Limnobaculum parvum*<br>*Lysobacter* sp. THG-D1<br>*Pseudoalteromonas* sp. 60CBH<br>*Pseudomonas stutzeri*<br>*Pseudomonas xanthomarina*<br>*Rugamonas rubra*<br>*Serratia plymuthica* PRI-2C<br>*Stenotrophomonas* sp. SO7.1<br>*Vibrio parahaemolyticus*<br>*Vibrio* sp.	cold fresh and brackish water<br>plant associated<br>arthropod endosymbiont<br>soil<br>spring water<br>animal gut<br>associated with seaweed<br>marine, ammonia oxidizer<br>animal gut<br>freshwater<br>soil, water, plant associated, hydrothermal vents<br>psychrophilic, deep marine<br>soil, denitrification<br>from marine ascidian (sea squirt - tunicate)<br>soil, rivers<br>soil, plant associated<br>copper mine drainage<br>brackish water, saltwater<br>saltwater
Hydrogenophilalia	*Hydrogenophilus thermoluteolus*	thermophilic chemolithoautotrophy
Oligoflexia	*Silvanigrella aquatica*	freshwater
Undetermined	Unknown bacterium endosymbiont<br>uncultured bacterium 183_29_M04	endosymbiont of arthropod<br>from coal bed core, salt marsh sediment, fish pathogen

**EUKARYA**<br>Animalia<br>Arthropoda	 <br>*Euryischia* sp. RDB-1999<br>*Gryllus bimaculatus*<br>*Linepithema humile*	 <br>arthropod (Australia)<br>arthropod (Africa)<br>arthropod (South America)
Apicomplexa	*Plasmodium cynomolgi*<br>*Plasmodium knowlesi*<br>*Plasmodium vivax*	parasite of arthropods, vertebrates<br>parasite of arthropods, vertebrates<br>parasite of arthropods, vertebrates
Archaeplastida<br>Chlorophyta<br><br><br><br><br><br><br>Streptophyta	 *Chlamydomonas* sp. UWO241<br>*Chlamydomonas noctigama*<br>*Chloropicon primus*<br>*Dunaliella salina*<br>Green microalgae<br>*Haematococcus lacustris*<br>*Monoraphidium neglectum*<br><br>Species from several plant families	 Antarctic<br>freshwater, terrestrial<br>marine microalga<br>halophilic microalga<br>marine<br>freshwater<br>freshwater<br><br>likely pollen or plant parts from glacier
Excavata<br>Euglenozoa	 *Parabodo caudatus*<br>*Parabodo nitrophilus*<br>*Rhynchomonas nasuta*<br>*Rhynchobodo* ATCC50359<br>*Dimastigella mimosa*<br>*Dimastigella trypaniformis*	 freshwater<br>freshwater<br>marine and freshwater<br>freshwater<br>soils<br>soils

Trypanosoma	*Leishmania donovani*<br>*Leishmania major*<br>*Leptomonas pyrrhocoris*<br>*Leptomonas seymouri*<br>*Trypanosoma carassii*<br>*Trypanosoma cruzi*<br>*Trypanosoma dionisii*<br>*Trypanosoma rotatorium*<br>*Trypanosoma* sp.<br>*Trypanosoma triglae*	arthropod, vertebrate<br>arthropod, vertebrate<br>arthropod<br>arthropod<br>annelids (leeches), fish (carp)<br>arthropods, vertebrates<br>arthropods, vertebrates<br>amphibians, annelids (leeches), arthropods<br>animals<br>annelids (leeches), fish (carp)
Fungi<br>Ascomycota	 *Saccharomyces* sp.<br>*Magnaporthe oryzae*<br>*Nannizzia gypsea*	 aquatic, soil, plants<br>plant pathogen<br>opportunist on animals, soil
Haptophyta	*Emiliania huxleyi*	coccolithophore, marine
Heterokonta (diatoms)	*Chaetoceros decipiens*<br>*Cylindrotheca closterium*<br>*Cylindrotheca* sp.<br>*Eucampia zodiacus*<br>*Nitzschia* sp.<br>*Navicula* sp.<br>*Navicula cryptotenella*<br>*Navicula arenaria*<br>*Pseudo-nitzschia americana*<br>*Skeletonema potamos*<br>*Skeletonema costatum*<br>*Thalassiosira lundiana*	polar marine diatom<br>marine diatom<br>marine diatom<br>marine diatom<br>marine diatom<br>aquatic diatom<br>benthic marine diatom<br>diatom, brackish water<br>planktonic marine diatom<br>diatom, brackish water and freshwater<br>marine diatom, brackish water<br>marine diatom

Table S2. Characteristics of organisms in western region of embayment. Taxa in the accretion ice representing the western section of the embayment (3,540 + 3,569 m sections), where characteristics could be determined.

Taxonomy	Species/Strain/Isolate	Characteristics (habitat, habit, functions)
**ARCHAEA**	*Halobacterium salinarum* R1	marine, halophilic
**BACTERIA**		
Actinobacteria	*Aeromicrobium* sp. *A1-2*	marine sediments, Antarctica
	Amycolatopsis sp. BJA-103	soil
	Auraticoccus monumenti	sandstone
	Brachybacterium saurashtrense	soil, halotolerant, plant associated
	Brachybacterium sp.	soil, animal feces
	Clavibacter michiganensis	soil, plant associated
	Corynebacterium timonense	soil, water, animal associated
	Kitasatospora setae	soil
	Gordonia sp. 1D	soil, thermotolerant
	Marmoricola scoriae	volcanic soil
	Micromonospora aurantiaca	soil, plant associated
	Micromonospora echinofusca	from marine sponge
	Micromonospora coxensis	sandy soil
	Micromonospora sp. L5	sediment, nitrate reduction, nitrogen fixation
	Micromonospora tulbaghiae	soil, plant associated
	Mycobacterium kansasii	fresh water, brackish water
	Mycobacterium sp. JS623	environmental

	Nocardia sp. CFHS0054<br>*Plantactinospora sp.* KBS50<br>*Pseudonocardia sp. HH130630-07*<br>*Rhodococcus aetherivorans*<br>*Rhodococcus opacus*<br>*Rhodococcus sp.* WB1<br>*Streptomyces albus*<br>*Streptomyces formicae*<br>*Streptomyces pluripotens*<br>*Streptomyces sampsonii*<br>*Streptomyces sp.* 3211<br>*Streptomyces sp. FR-008*<br>*Streptomyces sp.* GSSD-12<br>*Streptomyces sp.* Sge12<br>*Streptomyces sp. SM17*<br>*Streptomyces sp.* WAC00288<br>*Tessaracoccus sp.* T2.5-30<br>*Tsukamurella tyrosinosolvens*	nitrate reduction, environmental<br>marine<br>soil, arthropod associated<br>soil, bioreactor<br>soil, chemolithotrophic<br>soil<br>soil<br>soil, arthropod associated<br>mangrove soil<br>soil<br>soil<br>soil<br>soil<br>forest soil<br>from marine sponge (*Haliclona simulans*)<br>soil, alkalaitolerant<br>deep subsurface pyritic rock belt<br>water, air, soil
Cyanobacteria	*Arthrospira* sp. PCC 8005<br>uncultured cyanobacterium	alkaline and brackish water<br>marine
Gemmatimonadetes	*Gemmatirosa kalamazoonesis*	soil
Proteobacteria<br>Acidithiobacillia<br><br><br><br>α-proteobacteria	 *Acidithiobacillus ferrivorans* SS3<br>*Acidithiobacillus ferrooxidans ATCC 23270*<br>*Acidithiobacillus ferrooxidans ATCC 53993*<br><br>*Azospirillum brasilense* Sp245 (alpha)<br>*Azospirillum* sp. CFH 70021	 acidophilic, psychrotolerant, mining stream<br>bituminous coal mining stream, iron-oxidation<br>acidophilic, acid mine drainage, iron-oxidizing<br><br>soil, nitrogen fixation<br>hot spring water

	Phenylobacterium zucineum	aquifer, groundwater, sediments, soil, animals
	Rhodopseudomonas palustris BisA53	sediment
β-proteobacteria	*Achromobacter ruhlandii*	soil
	Achromobacter xylosoxidans	soil
	Bordetella bronchialis	environmental sources, animal associated
	Bordetella petrii	environmental sources, animal associated
	Massilia putida	wolfram mine
	Massilia sp. WG5	soil
	Methyloversatilis sp. *RAC08*	haptophyte associated
	Pandoraea oxalativorans	soil, chemolithoautotroph
	Pandoraea pnomenusa	soil, animal associated
	Paraburkholderia aromaticivorans	soil
	Paraburkholderia caledonica	soil
	Paraburkholderia sprentiae WSM5005	soil, plant associated
	Thiobacillus denitrificans ATCC 25259	groundwater, chemolithoautotrophy, sulfur-oxidation
δ-proteobacteria	*Sorangium cellulosum* So ce56	soil
γ-proteobacteria	*Pasteurella multocida*	soil, animal associated
	Serratia plymuthica PRI-2C	soil, plant associated
	Shigella sonnei	aquatic, animal gut
	Sulfurifustis variabilis	lake sediment, sulfur-oxidation, autotroph
**EUKARYA**		
Animalia		
Arthropoda	*Linepithema humile*	arthropod (South America)
Chordata	*Gavialis gangeticus*	reptile
Platyhelminthese	*Spirometra erinaceieuropaei*	tapeworm

Archaeplastida<br>Chlorophyta<br><br>Streptophyta	 *Monoraphidium neglectum*<br><br>Species from a few plant species	 freshwater green alga<br><br>likely pollen from glacier
Excavata<br>Trypanosoma	 *Trypanosoma grayi*	 amphibians, annelids, arthropods
Fungi<br>Ascomycota	 *Metarhizium brunneum* ARSEF 3297	 soil, arthropod associated

Table S3. Characteristics of organisms in the middle portion of the embayment. Taxa in the accretion ice representing the central section of the embayment (3,563 + 3,585 m sections), where characteristics could be determined. Data from refs. [1,2].

Taxonomy	Species/Strain/Isolate	Characteristics (habitat, habit, functions)
**BACTERIA**		
Actinobacteria	*Actinomyces georgiae*	soil, animal associated
	Actinomyces oris	soil, animal associated
	Actinomyces viscosus	soil, animal associated
	Agrococcus sp.	soil
	Arsenicicoccus bolidensis	lake sediment
	Arthrobacter flavus	psychrophilic, pond in McMurdo Dry Valley, Antarctica
	Arthrobacter sp. 01-Au-006/3	sediment, desiccation resistant
	Arthrobacter sp. V2M1	soil
	Atopobium parvulum	animal associated
	Bifidobacterium gallinarum	soil, animal associated
	Bifidobacterium sp.	soil, animal associated
	Blastococcus sp.	beach sediment
	Clavibacter michiganensis	soil, plant associated, psychrotolerant
	Corynebacterium durum	soil, water, plant associated, animal associated
	Corynebacterium sp.	soil, water, plant associated, animal associated
	Dermabacter sp.	soil, animal associated
	Frankia sp.	soil, plant associated, nitrogen fixation
	Frigoribacterium sp. GWS-SE-H243	soil

	Janibacter anopheles<br>*Janibacter* sp. MJ436<br>*Janibacter* sp. RC5-101<br>*Janibacter terrae*<br>*Klugiella* sp. Cr8-25<br>*Kocuria palustris*<br>*Kocuria* sp.<br>*Leifsonia kribbensis*<br>*Leifsonia* sp. MSL 07<br>Microbacteriaceae bacterium MIDF13<br>*Microbacterium* sp.<br>Micrococcaceae bacterium<br>*Micrococcus luteus*<br>*Micrococcus* sp.<br>*Mobilicoccus pelagius*<br>*Mycobacterium abscessus*<br>*Nesterenkonia lutea*<br>*Nesterenkonia sandarakina*<br>*Nocardioides* sp.<br>*Parascardovia denticolens*<br>*Phycicola gilvus*<br>*Rhodococcus* sp. WTZ-R2<br>*Rothia mucilaginosa*<br>*Rothia* sp.<br>*Sanguibacter* sp. ljh-8<br>*Streptomyces rimosus*<br>*Subtercola frigoramans*<br>*Thermobispora bispora*<br>*Yaniella soli*	midgut of arthropod (mosquito)<br>saline sediment, arid soil<br>hydrothermal marine sediment<br>soil near wastewater treatment plant<br>alpine glacier cryoconite<br>soil<br>water<br>soil<br>soil<br>soil<br>soil<br>air, dust<br>soil, dust, water, air<br>soil, dust, water, air<br>fish intestines<br>water, soil, dust<br>saline soil<br>arid soil<br>lakeside soil<br>animal gut, animal associated<br>marine, from seaweed<br>sediment, sludge<br>soil, dust, air, animal associated<br>soil, dust, air, animal associated<br>soil<br>soil<br>psychrophilic, cold groundwater<br>soil, thermophilic, decaying manure<br>soil, alkaliphilic

Bacteroidetes	*Anoxybacillus flavithermus*<br>*Bacteroides coprocola*<br>*Bacteroides* sp.<br>*Capnocytophaga granulosa*<br>*Flavobacterium johnsoniae*<br>*Flavobacterium* sp.<br>*Flavobacterium* sp. P-131<br>*Pedobacter steynii*<br>*Porphyromonas* sp.<br>*Prevotella denticola*<br>*Prevotella melaninogenica*<br>*Prevotella* sp.<br>*Sphingobacterium shayense*<br>*Sphingobacterium* sp. 0-1<br>*Sphingobacterium* sp. MOL-1	geothermal hot spring, thermotolerant<br>animal gut<br>animal gut<br>animal associated<br>soil, freshwater<br>Antarctica lake, halophilic, fish associated<br>cold water, fish associated<br>soil, Tibetan plateau<br>animal associated<br>animal associated<br>animal associated<br>animal associated<br>soil<br>lake, Tibetan plateau<br>psychrophilic, lake water
Cyanobacteria	*Anabaena azotica*<br>Antarctic cyanobacterium<br>*Brasilonema terrestre*<br>*Calothrix* sp. CCMEE 5085<br>*Chroococcidiopsis* sp.<br><br>*Geitlerinema* sp. CCALA 138<br>*Gloeocapsa* sp. CR_L16<br>*Leptolyngbya boryana*<br>*Leptolyngbya* sp.<br>*Lyngbya aestuarii*<br>*Lyngbya birgei*<br>*Leptolyngbya* sp. 22.A<br>*Microcoleus* sp.	soil, water, nitrogen fixation<br>Antarctic lake<br>soil<br>lake, Bolivian Andes<br>desiccation-tolerant, thermotolerant, psychrotolerant, halotolerant<br>soil, plant roots<br>aquatic, halophilic<br>freshwater, terrestrial<br>freshwater<br>marine intertidal<br>salt marches, freshwater, autotroph<br>freshwater<br>ponds, lakes, soil

	Microcoleus vaginatus<br>*Microcystis sp.*<br>*Nostoc flagelliforme*<br>*Nostoc muscorum*<br><br>*Nostoc* sp.<br><br>*Oscillatoria amoena*<br>*Oscillatoria lutea*<br>*Oscillatoria margaritifera*<br>*Oscillatoria nigro-viridis* PCC 7112<br>*Oscillatoria prolifera*<br>*Oscillatoria* sp.<br>*Oscillatoria* sp. 195-A20<br>*Oscillatoria* sp. MMG-2<br>Oscillatoriales cyanobacterium 2Dp86E<br>Oscillatoriales cyanobacterium IL-1.4<br>Phormidiaceae cyanobacterium CPER-KK1<br>*Phormidium autumnale*<br>*Phormidium* sp. NIVA-CYA 203<br>*Planktothricoides* sp.<br>soil crust cyanobacterium<br>*Synechococcus* sp. C9<br>*Thermosynechococcus* sp.<br>uncultured cyanobacterium<br>uncultured soil crust cyanobacterium	freshwater, dry puddles<br>freshwater<br>varying temperature and moisture conditions, oligotrophic<br>terrestrial and aquatic, desiccation resistant, nitrogen fixation<br>aquatic, oligotrophic, psychrotolerant, desiccation resistant, soil, rock, lakes, spring<br>marine, freshwater<br>freshwater, Sweden<br>marine<br>marine<br>freshwater reservoir<br>freshwater, marine<br>freshwater<br>freshwater<br>association with *Dyamena pumila* (white sea hydroid)<br>marine, estuary<br>soil<br>freshwater, benthic mat<br>lakewater, Spitzbergen, Svalbard<br>freshwater<br>soil, Antarctica<br>ammonium-replete hot spring<br>hot spring, thermophilic<br>freshwater<br>soil, rock
Deferribacteres	*Mucispirillum schaedleri*	animal intestinal tract (vertebrates and invertebrates)

Deinococcus-Thermus	*Deinococcus marmoris*<br><br>*Deinococcus radiodurans*	Antarctic marble, psychrophilic, lichen associated, radiation-resistant, desiccation resistant<br>soil, extremophile, radiation-resistant, psychrotolerant, desiccation resistant, acidotolerant
Firmicutes	*Amphibacillus* sp.<br>*Bacillus agaradhaerens*<br>*Bacillus cereus*<br>*Bacillus circulans*<br>*Bacillus cohnii*<br>*Bacillus decisifrondis*<br>*Bacillus halmapalus*<br>*Bacillus horikoshii*<br>*Bacillus horti*<br>*Bacillus megaterium*<br><br>*Bacillus* sp. 3LF 24T<br>*Bacillus* sp. 7327<br>*Bacillus* sp. 8SB<br>*Bacillus* sp. CCBAU 05776<br>*Bacillus* sp. CPB 7<br>*Bacillus* sp. MB63<br>*Bacillus* sp. NCIB 12289<br>*Carnobacterium mobile*<br>*Carnobacterium* sp. BM-8<br>*Clostridium perfringens*<br>*Clostridium rectum*<br>*Enterococcus columbae*<br>*Eubacterium cylindroides*<br>*Eubacterium tenue*	lake water, alkaliphilic, thermotoleant, desiccation resistant<br>lake water, alkaiphilic, alkalitolerant<br>soil, air, nitrate reduction, animal associated<br>soil, animal associated, plant associated<br>water, soil, sand, alkaliphilic<br>soil, decayed leaves<br>soil, alkaliphilic, alkalitolerant<br>fish liver, alkaliphilic, alkalitolerant<br>soil, alkaliphilic<br>soil, psychrotolerant, thermotolerant, Antarctic geothermal lake<br>soil<br>uranium mine wastewater<br>soil, spring, alkaliphilic<br>soil, plant associated<br>marine<br>soil, plant associated<br>soil, alkaiphilic<br>water, fish and crustacean associated<br>fish associated<br>marine sediment, soil, animal associated<br>soil, animal associated<br>soil, animal associated<br>soil, animal associated, intestinal<br>sludge, animal associated

	Flavonifractor plautii	soil, animal associated
	Geobacillus kaustophilus	deep sea sediment, thermophilic
	Jeotgalicoccus halotolerans	seafood, marine, halophilic
	Jeotgalicoccus sp. YIM KMY9-1	halophilic
	Lactobacillus animalis	soil, animal associated
	Lactobacillus curvatus	soil, animal associated
	Lactobacillus delbrueckii	soil, animal associated
	Lactobacillus fermentum	soil, animal associated, plant associated, thermotolerant
	Lactobacillus helveticus	soil, thermophilic, animal associated
	Lactobacillus rhamnosus	soil, animal associated
	Lactobacillus salivarius	soil, animal associated
	Lactococcus lactis	soil, animal associated, plant associated
	Marinococcus sp.	salt marsh, halophilic, chemolithoautotrophic
	Pediococcus claussenii	water, soil, plant associated
	Planomicrobium sp.	coastal sediment
	Planococcus maitriensis	cyanobacterial mat, Antarctica, psychrophilic
	Planococcus psychrotoleratus	cold desert soil, psychrotolerant
	Planococcus sp.	cold soil, psychrotolerant
	Planomicrobium koreense	glacier, seafood, psychrotolerant
	Planomicrobium psychrophilum	marine coastal sediment
	Planomicrobium sp. ISL-41	marine
	Planomicrobium sp. RCML-41	desert soil, radiation-resistant
	Robinsoniella peoriensis	soil, animal associated, intestinal
	Selenomonas sputigena	animal associated
	Sporosarcina sp. 4-76	soil
	Sporosarcina sp. LI4	soil
	Staphylococcus epidermidis	soil, dust, animal associated
	Streptococcus australis	soil, dust, animal associated
	Streptococcus constellatus	soil, dust, animal associated
	Streptococcus cristatus	soil, dust, animal associated
	Streptococcus dysgalactiae	soil, dust, animal associated

	Streptococcus equinus<br>*Streptococcus intermedius*<br>*Streptococcus mitis*<br>*Streptococcus mutans*<br>*Streptococcus sanguinis*<br>*Streptococcus* sp.<br>*Staphylococcus* sp.<br>*Streptococcus thermophilus*<br>*Tetragenococcus doogicus*<br>*Veillonella dispar*<br>*Veillonella parvula*	soil, dust, animal associated, intestinal<br>soil, dust, animal associated<br>soil, dust, animal associated<br>soil, water, acidotolerant, animal associated<br>soil, water, animal associated<br>soil, animal associated<br>soil<br>water, thermotolerant<br>seafood, halophilic<br>animal associated<br>animal associated
Fusobacteria	*Fusobacterium necrophorum*	animal associated
Proteobacteria<br>α-proteobacteria	 *Afifella marina*<br>*Amaricoccus sp.*<br>*Brevundimonas* sp. AKB-2008-JO46<br>*Brevundimonas* sp. MCS 35<br>*Caedibacter caryophilus*<br>*Caulobacter* sp. cau1<br>*Mesorhizobium loti*<br>*Methylobacterium fujisawaense*<br>*Methylobacterium* sp. iRIV1<br>*Paracoccus* sp. HMD3141<br>*Paracoccus* sp. J364<br>*Paracoccus* sp. JLT1284<br>*Paracoccus* sp. MC5-8<br>*Paracoccus* sp. sptzw33	 saline microbial mat<br>soil, sludge<br>soil, radiation resistant<br>aquatic, halotolerant<br>freshwater, symbiont of *Paramecium caudatum*<br>aquatic,<br>soil, plant associated, nitrogen fixation<br>soil, freshwater<br>soil, plant associated, animal associated<br>soil, denitrification, extremophilic, chemolithoautotroph<br>soil, animal associated, denitrification<br>marine, South China Sea<br>snow core, Dome A, East Antarctica<br>soil, extremophilic, denitrification

	Paracoccus versutus	soil, sludge, extremophilic, denitrification
	Paracoccus yeei	soil, water, animal associated
	Porphyrobacter sp.	marine
	Rhodobacter changlensis	psychrophilic, Himalayan snow
	Rhodobacter sp. RC5-103	water
	Sphingomonas dokdonensis	soil
	Sphingomonas sp. KT0216	soil, water, oligotrophic
	Sphingomonas sp.	soil, water
	Sulfitobacter sp.	marine
β-proteobacteria	*Acidovorax defluvii*	soil, sludge, denitrification
	Burkholderia sp.	soil, acidotolerant
	Burkholderia sp. Brij35	soil, sludge
	*Burkholderi*a sp. LD-11	soil
	Burkholderia sp. STM1424	soil
	Burkholderia vietnamiensis	soil, nitrogen fixation, metabolically versatile
	Caldimonas hydrothermale	thermal spring, thermophilic
	Caldimonas manganoxidans	hot spring, thermophilic, manganese oxidation
	Comamonadaceae bacterium	soil
	Comamonadaceae bacterium Gu-R-8	ancient subsurface sulfidic water
	Delftia acidovorans	soil, concentrates gold
	Denitrobacter sp. BBTR53	soil, sludge, denitrification
	Diaphorobacter sp. DNB7	soil, denitrification
	Herbaspirillum huttiense	water
	Herbaspirillum sp. B601	soil
	Neisseria flava	water, soil, sediment, animal associated
	Neisseria sp.	water, soil, sediment, animal associated
	Polaromonas sp.	groundwater, psychrophilic
	Sutterella sp.	soil, animal associated
δ-proteobacteria	Myxococcales bacterium	soil

ε-proteobacteria	*Campylobacter concisus*	water, animal associated
	Helicobacter sp.	water, soil, animal associated, acidotolerant
γ-proteobacteria	*Acinetobacter baumannii*	soil, water
	Acinetobacter calcoaceticus	soil, animal intestines
	Acinetobacter sp. QT15	soil, water, animal associated
	endosymbiont of arthropod	arthropod associated
	Enhydrobacter sp. KB3-12	freshwater lake
	Frateuria sp.	soil, plant associated, animal associated
	gamma proteobacterium enrichment culture clone BP44-5	marine
	Haemophilus haemolyticus	soil, water, wastewater, animal associated
	Halomonas boliviensis	soil, alkalitolerant, halophilic
	Halomonas sp. 2029	saltern, salt lake, halophilic
	Halomonas sp. AS-11	marine Arctic, halophilic
	Halomonas sp. G5 1-2	salt lake, halophilic
	Halomonas sp. NY93B	marine Arctic, halophilic, psychrotolerant
	Halomonas sp.	marine, halophilic
	Klebsiella sp. C611	soil, water, animal associated, plant associated
	Methylophaga sp.	marine, rock surfaces, halophilic
	Moraxella bovoculi	soil, dust, animal associated
	Moraxella osloensis	symbiont of nematode
	proteobacterium symbiont	isolated from arthropod
	Pseudoalteromonas sp. Ld19	deep-sea marine, psychrophilic
	Pseudomonas aeruginosa	soil, water, animal associated, plant associated
	Pseudomonas asplenii	water, plant associated
	Pseudomonas panipatensis	soil
	Pseudomonas putida	soil, water
	Pseudomonas sp. 14III/A01/008	alpine glacier
	Pseudomonas cf. stutzeri V4.MO.16	soil, water, denitrification

	Pseudomonas stutzeri<br>Pseudomonadaceae bacterium IZ2<br>*Psychrobacter faecalis*<br>*Psychrobacter frigidicola*<br>*Pseudomonas fluorescens*<br>*Psychrobacter immobilis*<br>*Pseudomonas* sp.<br>*Pseudomonas* sp. 47<br>*Pseudomonas* sp. 7325<br>*Pseudomonas* sp. B6_2008_<br>*Pseudomonas* sp. BSi20432<br>*Pseudomonas* sp. enrich. culture clone 13.1<br>*Pseudomonas* sp. Enrich. culture clone 23.2<br>*Pseudomonas* sp. SCT<br>*Pseudomonas* sp. SU19<br>*Pseudomonas* sp. SY6<br>*Pseudomonas* sp. WW6<br>*Pseudomonas* sp. ZR1-10<br>*Pseudomonas stutzeri*<br>*Pseudomonas xanthomarina*<br>*Psychrobacter frigidicola*<br>*Psychrobacter maritimus*<br>*Pseudomonas mendocina*<br>*Psychrobacter pulmonis*<br>*Psychrobacter* sp. Air226<br>*Psychrobacter* spp.<br>*Shigella* sp.<br>*Stenotrophomonas* sp. I_B14<br>*Xanthomonas fragariae*	soil, water, denitrification<br>soil, water<br>soil, water, animal associated<br>orthinogenic soil, Antarctica, psychrophilic<br>soil, water, plant associated<br>soil, water, fish associated, psychrophilic<br>soil, water<br>soil, animal associated, plant associated<br>Antarctica deep sea sediment<br>soil<br>sediment<br>soil, Ross Island, Antarctica<br>glacial ice, Svalbard<br>marine sediment<br>sediment<br>estuary, arsenite efflux pump<br>soil, plant associated<br>soil, plant associated<br>soil, animal associated<br>isolated from marine ascidian (tunicate), cold ocean<br>marine Antarctica, halophilic, psychrophilic<br>coastal sea ice, psychrotolerant<br>soil, aquifer, denitrification<br>sediments, seawater, permafrost, animal associated<br>soil, sea ice, fish gills and skin, Antarctic<br>marine, psychrophilic, halophilic<br>water, lakes, rivers, oceans<br>soil, plant associated, animal associated<br>soil, plant associated

Oligoflexia	*Bacteriovorax* sp.<br>*Bacteriovorax* sp. EPC3	marine<br>marine, Antarctica coast
Spirochaete	*Brachyspira pilosicoli*	water, feces, animal associated
Verrumicrobia	*Pedosphaera parvula*	soil
**EUKARYA**<br>Alveolata<br><br>Animalia<br>Arthropoda<br><br><br><br><br>Mollusca<br><br>Rotifera	 *Perkinsus marinus*<br><br><br>*Daphnia pulex*<br>*Entomobrya dorsosignata*<br>*Lepidocyrtus* sp.<br>*Sinella curviseta*<br><br>*Nutricola tantilla*<br><br>*Adineta vaga*	 marine bivalve pathogen<br><br><br>water flea, crustacean<br>leaf litter, springtail<br>wet soils, springtail<br>wet soils, springtail<br><br>deep water marine clam<br><br>freshwater
Archaeplastida<br>Chlorophyta	 *Microspora stagnorum*<br>*Pseudendoclonium akinetum*<br>*Pyramimonas tetrarhynchus*<br>*Zygnematales sp. M3006*	 freshwater<br>freshwater, terrestrial<br>freshwater<br>freshwater

Streptophyta	A few angiosperm species<br>Two gymnosperm species	likely pollen<br>likely pollen, both native to Himalayas
CIliophora	*Uroleptus pisces*	freshwater, planktonic
Excavata<br>Percoozoa<br><br>Trypanosoma	 *Naegleria gruberi*<br><br>*Trypanosoma cruzi*	 wet soil, freshwater, animal pathogen<br><br>animal parasite
Fungi<br>Ascomycota	 *Articulospora tetracladia*<br>*Boeremia exigua*<br>*Candida ontarioensis*<br>*Candida orthopsilosis*<br>*Cyberlindnera jadinii*<br>*Davidiella tassiana*<br>*Lecanora intumescens*<br>*Leptosphaeria doliolum*<br>*Mycosphaerellaceae* sp. CPC 12304<br>*Nigrospora* sp. SGSGf13<br>*Passalora perplexa*<br>*Peyronellaea glomerata*<br>*Phaeodothis winteri*<br>*Phaeosphaeria avenaria*<br>*Phaeosphaeria nodorum*<br>*Phaeosphaeria* sp. UZK<br>*Phoma infossa*	 aquatic<br>soil, plant associated<br>soil, plant associated<br>soil, animal associated, gut<br>soil, plant associated, animal associated<br>soil, plant associated (anamorph: *Cladosporium herbarum*)<br>rock, lichen associated<br>soil, plant associated<br>soil, plant associated<br>soil, plant associated<br>soil, plant associated<br>soil, marine, plant associated<br>soil, plant associated<br>soil, plant associated<br>soil, plant associated<br>soil, plant associated<br>soil, air, plant associated, animal associated

	Phoma macrostoma	soil, air, plant associated
	Phoma vasinfecta	soil, nematode associated, plant associated
	Phoma viburnicola	soil, air, plant associated, animal associated
	Saccharomyces cerevisiae	soil, water, plant associated
	Stagonosporopsis rudbeckiae	soil, plant associated
	Stagonosporopsis valerianellae	soil, plant associated
	Verticillium dahliae	soil, plant associated
Basidiomycota	*Cryptococcus neoformans*	soil, animal associated, plant associated
	Cryptococcus sp. ATT123	soil
	Dioszegia rishiriensis	soil
	Malassezia pachydermatis	soil, animal associated
	Rhodotorula lamellibrachiae	marine animal associated, 1100 m depth
	Sakaguchia dacryoidea	marine, alkalitolerant, Antarctic seawater 4000 m, glacial meltwater
	Sistotrema brinkmannii	soil, plant associated, fungus associated
Zygomycota	*Gongronella* sp. xt-2009	soil, plant associated
Heterokonta	*Aphanomyces euteiches*	freshwater, plant associated
	Botrydiopsis constricta	terrestrial, Antarctica
	Halosiphon tomentosus	marine
	Stephanodiscus sp. FHTC11	freshwater diatom, lake, river
	Stephanodiscus sp. KHR001	freshwater diatom, reservoir
	uncultured labyrinthulid	marine slime nets

Table S4. Characteristics of organisms in the eastern portion of the embayment. Taxa in the accretion ice representing the eastern section of the embayment (3,585 m section), where characteristics could be determined.

Taxonomy	Species/Strain/Isolate	Characteristics (habitat, habit, functions)
**BACTERIA**<br>Actinobacteria	*Microbacterium* sp.	soil, water
Bacteroidetes	*Arachidicoccus* sp.<br>*Chitinophaga caeni*<br>*Chitinophaga* sp.<br>*Salinibacter ruber*	soil, plant associated<br>soil, sediment<br>soil<br>aquatic, halophilic
Cyanobacteria	*Phormidium* sp.	aquatic
Firmicutes	*Bacillus mojavensis*<br>*Bacillus* sp.<br>*Bacillus subtilis*<br>*Bacillus thuringiensis*<br>*Bhargavaea* sp.	desert soil<br>soil, thermotolerant<br>soil<br>soil, aquatic, arthropod associated, animal gastrointestinal<br>deep sea sediments, coastal sand
Planctomyces	*Planctomyces* sp.	marine
Proteobacteria<br>α-proteobacteria<br><br>β-proteobacteria	 *Phycorickettsia trachydisci*<br><br>*Achromobacter insolitus*	 algal endosymbiont<br><br>soil, water, animal associated

	Achromobacter sp.	soil
	Achromobacter xylosoxidans	wet soil, aquatic, animal associated
	Acidovorax cattleyae	soil, plant associated
	Acidovorax citrulli	soil, plant associated
	Acidovorax sp.	soil
	Advenella kashmirensis	soil
	Advenella sp.	soil
	Alcaligenes aquatilis	salt marsh sediments
	Aquabacterium olei	soil, water
	Azoarcus sp.	soil, freshwater
	Bordetella parapertussis	airborne, soil, animal associated
	Bordetella pertussis	airborne, soil, animal associated
	Crenobacter sp.	soil, water
	Dechloromonas hortensis	water, karst cave
	Dechloromonas sp.	soil, water
	Delftia sp.	soil, freshwater, crustaceans
	Janthinobacterium agaricidamnosum	soil, psychrotolerant, Antarctic
	Limnohabitans sp.	freshwater, planktonic
	Melaminivora sp.	soil, sediment
	Methylopumilus planktonicus	freshwater, planktonic, methylotrophic
	Methylophilus sp.	soil, plant associated
	Methylopumilus turicensis	freshwater, planktonic, methylotrophic
	Methylotenera mobilis	freshwater, planktonic, methylotrophic
	Ottowia oryzae	soil, sediment, plant associated
	Pelomonas sp.	aquatic
	Polaromonas glacialis	alpine glacier cryoconite, psychrophilic
	Pusillimonas ginsengisoli	soil
	Pusillimonas sp.	soil, mud, sand, psychrotolerant
	Rhizobacter gummiphilus	soil, plant associated
	Simplicispira suum	air, sediment, dust
	Undibacterium sp.	freshwater, soil

	Variovorax sp.<br>*Zoogloea* sp.	soil, aquatic<br>sediments
δ-proteobacteria	*Pelobacter* sp.	marine sediments
γ-proteobacteria	*Baumannia cicadellinicola*<br>*Klebsiella sp.*<br>*Marinimicrobium sp.*<br>*Nitrincola alkalilacustris*<br>*Nitrincola schmidtii*<br>*Pantoea sp.*<br>*Pseudocitrobacter anthropic*<br>*Pseudomonas bohemica*<br>*Pseudomonas brassicacearum*<br>*Pseudomonas brenneri*<br>*Pseudomonas marginalis*<br>*Pseudomonas monteilii*<br>*Pseudomonas putida*<br>*Pseudomonas savastanoi*<br>*Pseudomonas* sp.<br>*Pseudomonas thivervalensis*<br>*Psychrobacter* sp.<br><br>*Sodalis* sp.	endosymbiont of arthropod<br>soil, water, plant associated, animal associated<br>soil, aquatic, tidal flat sediment, alkaliphilic. halotolerant<br>soil, aquatic, alkaliphilic, halotolerant<br>soil, aquatic, alkaliphilic, halotolerant<br>soil, freshwater, animal associated, plant associated<br>animal gastrointestinal<br>soil, animal associated<br>soil, plant associated<br>water<br>soil, plant associated<br>soil, animal associated<br>soil, water, plant associated<br>moist soil, airborne, plant associated<br>soil, plant associated, animal associated<br>soil, plant associated<br>aquatic, psychrophilic, psycchotolerant, halotolerant,<br>Antarctica<br>endosymbiont of arthropod
**EUKARYA**<br>Alveolata<br>CIliophora	 <br>*Corlissina maricaensis*<br>*Trachelolophos quadrinucleatus*	 <br>ciliated protozoan, brackish water<br>protozoan, marine sandy intertidal

Amoebozoa	*Difflugia bacillariarum*<br>*Difflugia hiraethogii*<br>*Difflugia lanceolata*<br>*Difflugia oblonga*	freshwater<br>freshwater<br>freshwater<br>freshwater marsh
Animalia<br>Arthropoda<br><br>Choanoflagellatea	 <br><br>uncultured Choanoflagellida	 <br><br>unicellular animal, marine, brackish water, freshwater
Fungi<br>Ascomycota	 *Arthrocatena tenebrio*<br>*Ascochyta sorghi*<br>*Boeremia exigua*<br>*Didymella macrostoma*<br>*Epicoccum nigrum*<br>*Epicoccum sorghinum*<br>*Neoascochyta graminicola*<br>*Nothophoma quercina*<br>*Phoma* sp.<br>*Rachicladosporium eucalypti*<br>*Readerielliopsis fuscoporiae*<br>*Stagonosporopsis cucurbitacearum*	 rock inhabiting<br>soil, plant associated<br>soil, plant associated<br>soil, plant associated<br>soil, plant associated<br>soil, plant associated<br>soil, plant associated<br>soil, plant associated<br>soil, plant associated<br>soil, plant associated<br>soil, plant associated<br>soil, plant associated
Heterokonta (diatoms)	*Phaeodactylium stadleri*	marine diatom
Stramenopile	*Chrysopodocystis socialis*	marine

Table S5. Characteristics of organisms in the western main basin of Lake Vostok. Taxa in the accretion ice representing the western edge of the main basin (3,606 + 3,621 m sections), where characteristics could be determined. Data from refs. [1,2].

Taxonomy	Species/Strain/Isolate	Characteristics (habitat, habit, functions)
**BACTERIA**		
Actinobacteria	*Clavibacter michiganensis*	Soil, plant associated
	Micrococcus sp.	soil, water
	Mycobacterium marinum	marine, freshwater, fish associated
Firmicutes	*Bacillus clausii*	soil
	Lactococcus lactis	animal associated, plant associated
	Sporosarcina globispora	soil, river water, psychrophilic
	Staphylococcus sp.	soil, halotolerant, desiccation resistant
Proteobacteria		
α-proteobacteria	*Brevundimonas diminuta*	Soil, water
	Brevundimonas sp. AKB-2008-KU11	soil, freshwater
	Mycoplana sp.	soil
	Subaequorebacter tamlense	beach sediment
β-proteobacteria	*Burkholderia cepacia*	soil, water
	Burkholderia sp. CV4.4.3R1	soil
	Comamonas sp. BF-3	soil
	Delftia acidovorans	soil, rock
	Herbaspirillum sp.	soil
	Lautropia mirabilis	animal associated
	Thiobacillus sp.	soil, autotroph

γ-proteobacteria	*Citrobacter* sp.<br>*Enterobacter* sp.<br>*Escherichia* sp.<br>*Moraxella bovoculi*<br>*Pseudomonas putida*<br>*Pseudomonas* sp.<br>rainbow trout intestinal bacterium T1<br>*Rheinheimera* sp.<br>*Shigella* sp.<br>*Vibrio* sp.<br>*Xanthomonas fragariae*	soil, freshwater, sediment<br>animal gut<br>animal gut, soil, water<br>animal associated<br>soil<br>soil, freshwater<br>animal gut<br>lake water<br>animal gut, soil, water<br>freshwater lake sediment<br>soil, plant associated
Undetermined	lobster gut bacterium ABHa3	crustacean associated
**EUKARYA**<br>Archaeplastida<br>Streptophyta	 <br>Two sequences from two plan families	 <br>likely pollen
Fungi<br>Ascomycota	 *Babjeviella inositovora*<br>*Candida tropicalis*<br>*Cladosporium cladosporioides*<br>*Coniosporium apollinis*<br>*Cyathicula microspore*<br>*Davidiella tassiana*<br>*Medeolaria farlowii*<br>*Millerozyma farinose*	 soil<br>soil, water, animal gut, halotolerant<br>psychrophilic, desiccation resistant, soil, water, air<br>rock (marble)<br>soil, water<br>soil, plant associated<br>soil, water, plant associated<br>halotolerant, osmotolerant

	Ogataea thermomethanolica<br>*Phaeosphaeria avenaria*	thermotolerant, methylotrophic<br>soil, plant associated
Basidiomycota	*Bullera taiwanensis*<br>*Geastrum sessile*	soil, plant associated<br>soil

Table S6 Species and isolates listed by characteristics. Characteristics (where they could be determined) and isolate sources for species closest to those in the Vostok ice core sections, with taxon designations.

Basal (3,501 + 3,520 m)

Marine estuary – *Mycobacterium* sp. (Actinobacteria)

Deep ocean – *Euzebya* sp. (Actinobacteria)

Antarctic quartz – *Aequorivita sublithinicola* (Actinobacteria)

Alpine glacier cryoconite – *Pedobacter cryoconitis* (Bcteroidetes)

Animal gastrointestinal – *Balutia coccoides* (Firmicutes), *Caecibacter massiliensis* (Firmicutes), *Lactobacillus plantarum* (Firmicutes), *Megasphaera hexanoica* (Firmicutes), *Negativeicoccus massiliensis* (Firmicutes), *Enterobacter* sp. (Gammaproteobacteria), *Cellvibrio* sp. (Gammaproteobacteria)

Animal pathogen – *Trypanosoma* sp. (Excavata)

Annelids – *Pseudarcicella* sp. (Bacteroidetes)

Antarctica – *Paenisporosarcina* sp. (Firmicutes)

Antarctic lichen – *Hymenobacter* sp. (Bacteroidetes)

Antarctic saline lake - *Flavobacterium* sp. (Bacteroidetes)

Antarctic soil – *Sphingobacterium* sp. (Bacteroidetes)

Apicomlexa – 3 *Plasmodium* species

Arsenic metabolism– *Acidiphilium miltivorum* (Alphaproteobacteria)

Arthropod symbiont – *Buchnera aphidicola* (Gammaproteobacteria)

Brown algae-associated – *Mariniflexile* sp. (Bacteroidetes)

Chlorophyta (green algae) – 7 species (Antarctic, aquatic, marine, soil)

Chromium metabolism - *Acidiphilium cryptum* (Alphaproteobacteria)

Diatoms (Heterokonta) – 12 species (aquatic and marine)

Diatom-associated – *Kordia* sp. (Bacteroidetes)

Euglenozoa – 6 species (aquatic, marine, soil)

Fish associated– *Flavobacterium branchiophilum* (Bacteroidetes), *Flavobacterium columnare* (Bacteroidetes), *Flavobacterium psychrophilum* (Bacteroidetes), *Flavobacterium* sp. (Bacteroidetes), *Raoultella planticola* (Betaproteobacteria)

Fish/Amphibian associated – *Elizabethkinia meningosepta* (Bacteroidetes)

Fungi – 3 species (Ascomycota)

Glacier (Tibet) – *Massilia glaciei* (Betaproteobacteria)

Halophilic/halotolerant - *Dunaliella salina* (Chlorophyta), *Halorubrum trapanicum* (Archaea)

Haptophyte - *Emiliania huxleyi* (marine coccolithophore)

Hydrothermal vent – *Lysobacter* sp. (Gammaproteobacteria)

Iron metabolism – *Acidiphilium cryptum* (Alphaproteobacteria), *Ferribacterium* sp. (Betaproteobacteria), *Ferriphaselus amnicola* (Betaproteobacteria),

Marine hydrothermal vent – *Hippea maritima* (Deltaproteobacteria)

Mussel farm - *Echinicola vientamensis* (Bacteroidetes)

Pacific oyster – *Flavobacterium crassostreae* (Bacteroidetes)

Peat bog/permafrost – *Paludisphaera borealis* (Planctomycete)

Penguin feces - *Flavobacterium kingsejongi* (Bacteroidetes)

Psychrophilic/psychrotolerant - *Cryobacterium* sp. (Actinobacteria), *Hathewaya histolytica* (Actinobacteria), *Pedobacter cryoconitis* (Bacteroidetes), *Paenisporosarcina* sp. (Firmicutes), *Rhodoferax* sp. (Betaproteobacteria), *Pseudoalteromonas* sp. (Gammaproteobacteria)

Radiation tolerant – *Kineococcus radiotolernas* (Betaproteobacteria)

Rock – *Delftia acidovorans* (Betaprtoeobacteria), *Massilia buxea* (Betaproteobacteria)

Sand dollar associated – *Cyclobacterium marinum* (Bacteroidetes)

Sea squirt associated – *Psuedomonas xanthomarina* (Gammaproteobacteria)

Sea urchin associated – *Echinicola strongylocenroti* (Bacteroidetes)

Seaweed associated – *Gilvimarinus* sp. (Gammaprotebacteria)

Sulfur metabolism – *Desulfococcus multivorans* (Betaproteobacteria), *Sulfuriferula*

thiophila (Betaproteobacteria), *Desulfurella acetovorans* (Deltaproteobacteria), *Desulfobacterium* sp. (Deltaproteobacteria), *Hippea maritima* (Deltaproteobacteria)

Thermophilic/thermotolerant - *Thauera hydrothermalis* (Betaproteobacteria), *Desulfurella acetivorans* (Delta), *Hydrogenophilus thermoluteolus* (Hydrogenophilalia)

Tidal flat - *Flavobacterium* sp. (Bacteroidetes)

Tidal flat – *Gramella* sp. (Bacteroidetes)

Wolfram (manganese tungstate) – *Massilia pudita* (Betaproteobacteria)

Western section of embayment (3,540 + 3,569 m)

Animal gastrointestinal - *Brachybacterium* sp. (Actinobacteria), *Shigella sonnei* (Gammaproteobacteria)

Animal pathogen – *Trypanosoma* sp. (Excavata)

Chlorophyta (green alga) - *Monoraphidium neglectum* (Archaeplastida)

Deep subsurface pyritic rock - *Tessaracoccus* sp. (Actinobacteria)

Fungi - *Metarhizium brunneum* (Ascomycota)

Halophilic/halotolerant - *Halobacterium salinarum* (Archaea), Brachybacterium saurashtrense (Actinobacteria)

Haptophyte associated - *Methyloversatilis* sp. (Betaproteobacteria)

Hot spring - *Azospirillum* sp. (Alphaproteobacteria)

Iron metabolism - *Acidithiobacillus ferrooxidans* (Actinoproteobacteria)

Marine sponge - *Micromonospora echinofusca* (Actino), *Streptomyces* sp. (Actinobacteria)

Psychrophilic/psychrotolerant - *Acidithiobacillus ferrivorans* (Acidithiobacillia)

Sandstone - *Auraticoccus monumenti* (Actinobacteria)

Sulfur metabolism - *Thiobacillus denitrificans* (Betaproteobacteria), *Sulfurifustis variabilis* (Gammaproteobacteria)

Thermophilic/thermotolerant - *Gordonia* sp. (Actinobacteria)

Volcanic soil - *Marmoricola scoriae* (Actinobacteria)

Middle Section of Embayment (3,563 + 3,585 m)

Alpine glacier - *Pseudomonas* sp. (Gammaproteobacteria)

Alpine glacier cryoconite - *Klugiella* sp. (Actinobacteria)

Ancient subsurface sulfidic water - *Caldimonas manganoxidans* (Betaproteobacteria)

Andes lake - *Calothrix* sp. (Cyanobacteria)

Animal gastrointestinal - *Janibacter anopheles* (Actinobacteria), *Parascardovia denticolens* (Actinobacteria), *Bacteroides coprocola* (Bacteroidetes), *Bacteroides* sp. (Bacteroidetes), *Mucispirillum schaedleri* (Deferribacteres), *Streptococcus equinus* (Firmicutes), *Acinetobacter calcoaceticus* (Gammaproteobacteria), *Brachyspira pilosicoli* (Spirochaete), *Candida orthopsilosis* (Ascomycota)

Animal parasite - *Trypanosoma* sp. (Excavata)

Antarctica – rock crust cyanobacterium, *Planococcus maitriensis* (Firmicutes), *Paracoccus* sp. (Alphaproteobacteria), *Psychrobacter frigidicola* (Gammaproteobacteria), *Psychrobacter* sp. (Gammaproteobacteria)

Antarctic coast - *Bacteriovorax* sp. (Oligoflexia)

Antarctic deep sea (4000 m) - *Sakaguchia dacryoidea* (Basidiomycota)

Antarctic deep sea sediment - *Pseudomonas* sp. (Gammaproteobacteria)

Antarctic lake - *Flavobacterium* sp. (Bacteroidetes)

Antarctic marble - *Deinococcus marmoris* (Deinococcus-Thermus)

Arctic - *Halomonas* sp. (Gammaproteobacteria)

Deep sea - *Pseudoalteromonas* sp. (Gammaproteobacteria)

Deep sea sediment - *Geobacillus kaustophilus* (Firmicutes)

Fish gastrointestinal - *Mobilicoccus pelagius* (Actinobbacteria), *Flavobacterium* sp. (Bacteroidetes)

Fish liver - *Bacillus horikoshii* (Firmicutes)

Fish/crustaceans - *Carnobacterium mobile* (Firmicutes), *Carnobacterium* sp. (Firmicutes), *Psychrobacter immobilis* (Gammaproteobacteria)

Geothermal hot spring - *Anoxybacillus flavithermus* (Bacteroidetes), Synecococcus sp. (Cyanobacterium), Thermosynecococcus sp. (Cyanobacterium)

Glacial ice, Svalbard - *Pseudomonas* sp. (Gammaproteobacteria)

Glacial meltwater - *Sakaguchia dacryoidea* (Basidiomycota)

Glacier - *Planomicrobium koreense* (Firmicutes)

Halophilic/halotolerant - *Flavobacterium* sp. (Bacteroidetes), *Chroococcidiopsis* sp. (Cyanobacteria), *Gloeocapsa* sp. (Cyanobacteria), *Jeotgalicoccus halotolerans* (Firmicutes), *Jeotgalicoccus* sp. (Firmicutes), *Marinococcus* sp. (Firmicutes), *Tetragenococcus doogicus* (Firmicutes), *Brevundimonas* sp. (Alphaproteobacteria), *Halomonas boliviensis* (Gammaproteobacteria), *Halomonas* sp. (Gammaproteobacteria), *Methylophaga* sp. (Gammaproteobacteria), *Psychrobacter* sp. (Gammaproteobacteria)

Diatoms – 6 species, aquatic, marine, Antarctic (Heterokonta)

Himalayan snow - *Rhodobacter changlensis* (Alphaproteobacteria)

Hot spring - *Caldimonas manganoxidans* (Alphaproteobacteria)

Lichen associated - *Deinococcus marmoris* (Deinococcus-Thermus)

Marine Arctic - *Halomonas* sp. (Gammaproteobacteria)

Marine hydrothermal sediment - *Janibacter* sp. (Actinobacteria)

Marine sea hydroid associated - Oscillatoriales (Cyanobacteria)

Marine tunicate associated - *Pseudomonas xanthomarina* (Gammaproteobacteria)

McMurdo Dry Valley, Antarctica - *Arthrobacter flavus* (Actinobacteria)

Psychrophilic/psychrotolerant - *Arthrobacter flavus* (Actinobacteria), *Clavibacter michiganensis*(Actinobacteria), *Subtercola frigoramans* (Actinobacteria), *Sphingobacterium* sp. (Bacteroidetes), *Chroococcidiopsis* sp. (Cyanobacteria), *Nostoc* sp. (Cyanobacteria), *Deinococcus marmoris* (Deinococcus-Thermus), *Deinococcus radiodurans* (Deinococcus-Thermus), *Bacillus megaterium* (Firmicutes), *Planococcus maitriensis* (Firmicutes), *Planococcus psychrotoleratus* (Firmicutes), *Planococcus* sp. (Firmicutes), *Planomicrobium koreense* (Firmicutes), *Rhodobacter changlensis* (Alphaproteobacteria), *Polaromonas* sp. (Betaproteobacteria), *Halomonas* sp. (Gammaproteobacteria), *Pseudoalteromonas* sp.

(Gammaproteobacteria), *Psychrobacter frigidicola* (Gammaproteobacteria), *Psychrobacter immobilis* (Gammaproteobacteria), *Psychrobacter* sp. (Gammaprotebacteria)

Rock – rock crust cyanobacterium

Ross Island, Antarctica - *Pseudomonas* sp. (Gammaproteobacteria)

Seaweed - *Phycicola gilvus* (Actinobacteria)

Symbiont of nematode - *Moraxella osloensis* (Gammaproteobacteria)

Symbiont of Paramecium - *Caedibacter caryophilus* (Alphaproteobacteria)

Svalbard - *Phormidium* sp. (Cyanobacterium)

Thermal stream - *Caldimonas hydrothermale* (Betaproteobacteria)

Thermophilic/thermotolerant - *Thermobispora bispora* (Actinobacteria), *Anoxybacillus flavithermus (*Bacteroidetes), *Thermosynechococcus* sp. (Cyanobacteria), *Bacillus megaterium* (Firmicutes), *Geobacillus kaustophilus* (Firmicutes), *Lactobacillus fermentum* (Firmicutes), *Lactobacillus helveticus* (Firmicutes), *Streptococcus thermophilus* (Firmicutes), *Caldimonas hydrothermale* (Betaproeobacteria), *Caldimonas manganoxidans* (Betaproteobacteria)

Tibetan plateau lake - *Pedobacter steynii* (Bacteroidetes)

Tibetan plateau soil – *Sphingobacterium* sp. (Bacteroidetes)

Eastern section of embayment (3,585 m)

Algal symbiont - *Phycorickettsia trachydisci* (Alphaproteobacteria)

Alpine glacier cryoconite - *Polaromonas glacialis* (Betaproteobacteria)

Animal gastrointestinal - *Bacillus thuringiensis* (Firmicutes)

Antarctica - *Janthinobacterium agaricidamnosum* (Betaproteobacteria), *Psychrobacter* sp. (Gammaproteobacteria)

Brackish water - *Corlissina maricaensis* (Ciliopora)

Ciliated protozoan - *Corlissina maricaensis* (Ciliophora), *Trachelolophos quadrinucleatus* (Ciliophora)

Crustacean associated - *Delftia* sp. (Betaproteobacteria)

Cave (karst, water) - *Dechloromonas hortensis* (Betaproteobacteria)

Coastal sand - *Bhargavaea* sp. (Firmicutes)

Deep sea sediment - *Bhargavaea* sp. (Firmicutes)

Endosymbiont of arthropod - *Sodalis* sp. (Gammaproteobacteria)

Freshwater marsh - *Difflugia oblonga* (Amoebozoa)

Halophilic/halotolerant - *Salinibacter ruber* (Bacteroidetes), *Marinimicrobium* sp. (Gammaproteobacteria), *Nitrincola alkalilacustris* (Gammaproteobacteria), *Nitrincola schmidtii* (Gammaproteobacteria), *Psychrobacter* sp. (Gammaproteobacteria)

Marine brackish water - uncultured Choanoflagellida (Choanoflagellatea)

Marine diatom - *Phaeodactylium stadleri* (Heterokonta)

Marine sandy intertidal - *Trachelolophos quadrinucleatus* (Ciliophora)

Methylotrophic - *Methylopumilus planktonicus* (Betaproteobacteria), *Methylopumilus turicensis* (Betaproteobacteria), *Methylotenera mobilis* (Betaproteobacteria)

Psychrophilic/psychrotolerant - *Janthinobacterium agaricidamnosum*

(Betaproteobacteria), *Polaromonas glacialis* (Betaproteobacteria), *Pusillimonas* sp. (Betaprotebacteria), *Psychrobacter* sp. (Gammaprotebacteria)

Rock inhabiting - *Arthrocatena tenebrio* (Ascomycota)

Salt marsh sediments - *Alcaligenes aquatilis* (Betaproteobacteria)

Thermophilic/thermotolerant - *Bacillus* sp. (Firmicutes)

Tidal flat sediment - *Marinimicrobium* sp. (Gammaproteobacteria)

Main Basin (3,606 + 3,621 m)

Animal gastrointestinal - *Enterobacter* sp. (Gammaproteobacteria), *Escherichia* sp.

(Gammaproteobacteria), rainbow trout intestinal bacterium T1 (Gammaproteobacteria), *Shigella* sp. (Gammaproteobacteria), *Candida tropicalis* (Ascomycota)

Beach sediment - *Subaequorebacter tamlense* (Alphaproteobacteria)

Crustacean associated - lobster gut bacterium ABHa3 (phylum undetermined)

Fish associated - *Mycobacterium marinum* (Actinobacteria)

Halophilic/halotolerant - *Staphylococcus* sp. (Firmicutes), *Candida tropicalis* (Ascomycota), *Millerozyma farinose* (Ascomycota)

Lake - *Rheinheimera* sp. (Gammaproteobacteria)

Lake sediment - *Citrobacter* sp. (Gammaproteobacteria), *Vibrio* sp. (Gammaprotebacteria)

Methylotrophic - *Ogataea thermomethanolica* (Ascomycota)

Psychrophilic/psychrotolerant - *Sporosarcina globispora* (Firmicutes), *Cladosporium cladosporioides* (Ascomycota)

River - *Sporosarcina globispora* (Firmicutes)

Rock - *Delftia acidovorans* (Betaproteobacteria), *Coniosporium apollinis* (Ascomycota)

Thermophilic/thermotolerant - *Ogataea thermomethanolica* (Ascomycota)

Table S7. Enumeration of sequence reads and unique sequences. Each unique sequence represents a separate species or strain.

GLACIAL (2,149m)

TAXON	READS	UNIQUE SEQs.	CHARACTERS[a]
**BACTERIA**			
Cyanobacteria	97	12	au, ps

BASAL (3,501 + 3.520 m)

TAXON	READS	UNIQUE SEQs.	CHARACTERS[a]
**BACTERIA**			
Actinobacteria	114	47	aq, al, de, ha, he, ma, nf, no, ps, so, th
Bacteroidetes	5708	86	al, an, aq, au, ps, so, th
Cyanobacteria	530	2	au, ps, th
Elusimicrobia	3	1	aq, ma, so
Firmicutes	61	37	ae, an, au, ha, ma, ps, th
Gemmatimonadetes	1	1	aq, so

Planctomycetes	1	1	an, aq, au, ma, nr
Proteobacteria			
Alphaproteobacteria	17	11	aa, al, aq, au, ha, ma, me, nr, ps, sm, so, th
Betaproteobacteria	3307	129	ae, aq, au, nf, me, no, nr, ps, am, th
Deltaproteobacteria	30	11	ae, an, aq, au, ha, ma, am, so
Epsilonproteobacteria	20	5	aa, ae, an, au, th
Gammaproteobacteria	50	37	aa, ae, an, aq, au, ha, ic, ma, nf, no, nr, ps, sm, so, th
Hydrogeophilales	4	1	aq, au, th
Oligoflexia	5	5	aq
Spirochaetes	3	3	aa, he
**ARCHAEA**	1	1	he, ma, ps, so
**EUKARYA**			
Archaeplastida			
Chlorophyta	330	9	au, ps
Streptophyta	93	23	au
Chromalveolata	1172	21	aq, au, ps, he
Excavata	21	18	aq, he
Haptophyta	3	1	ma, so
Opisthokonta			
Animalia			
Arthropoda	3	3	aq, he, ps
Fungi			
Ascomycota	84	21	aq, he, ps, so

WEST REGION OF EMBAYMENT (3540 + 3569 m)

TAXON	READS	UNIQUE SEQs.	CHARACTERS
**BACTERIA**			
Actinobacteria	362	40	aq, al, de, ha, he, ma, nf, no, ps, so, th
Cyanobacteria	3	2	au, ps, th
Gemmatimonadetes	1	1	aq, so
Proteobacteria			
Acidithiobacillia	2	2	aa, ae, aq
Alphaproteobacteria	4	3	aa, al, aq, au, ha, ma, me, nr, ps, sm, so, th
Betaproteobacteria	1309	33	ae, aq, au, nf, me, no, nr, ps, am, th
Deltaproteobacteria	5	2	ae, an, aq, au, ha, ma, am, so
Gammaproteobacteria.	122	15	aa, ae, an, aq, au, ha, ic, ma, nf, no, nr, ps, sm, so, th
**ARCHAEA**	2	2	ha, he, ma, ps, so
**EUKARYA**			
Archaeplastida			
Streptophyta	4	3	au
Excavata	1	1	aq, he
Opisthokonta			
Animalia			
Arthropoda	1	1	aq, he, ps
Fungi			
Ascomycota	1	1	aq, he, ps, so

MIDDLE REGION OF EMBAYMENT (3,563 + 3,585 m)

TAXON	READS	UNIQUE SEQs.	CHARACTERS[a]
**BACTERIA**			
Acidobacteria	nd	2	ac, aq, he, so
Actinobacteria	nd	228	aq, al, de, ha, he, ma, nf, no, ps, so, th
Bacteroidetes/Chlorobi	nd	85	al, an, aq, au, ps, so, th
Chloroflexi	nd	1	ae, au, th
Cyanobacteria	nd	228	au, ps, th
Deferribacteres	nd	1	aa, an, he
Deinococcus/Thermus	nd	5	ae, an, he, th
Fibrobacteres	nd	1	aa, ae, he
Firmicutes	nd	602	ae, an, au, ha, ma, ps, th
Fusobacteria	nd	10	aa, an, he
Planctomycetes	nd	6	an, aq, au, ma, nr
Proteobacteria			
Alphaproteobacteria	nd	91	aa, al, aq, au, ha, ma, me, nr, ps, sm, so, th
Betaproteobacteria	nd	105	ae, aq, au, nf, me, no, nr, ps, am, th
Deltaproteobacteria	nd	10	ae, an, aq, au, ha, ma, am, so
Epsilonproteobacteria	nd	6	aa, ae, an, au, th
Gammaproteobacteria	nd	254	aa, ae, an, aq, au, ha, ic, ma, nf, no, nr, ps, sm, so, th
Spirochaetes	nd	8	aa, he
Tenericutes	nd	4	aa, he
Verrucomicrobia	nd	3	aa, ae, aq, he, so

**ARCHAEA**	nd	2	he, ma, ps, so
**EUKARYA**			
Amoebozoa	nd	1	aq, he
Archaeplastida			
Chlorophyta	nd	10	au, ps
Rhodophyta	nd	1	au, ps
Streptophyta	nd	66	au
Chromalveolata	nd	12	aq, au, ps, he
Excavata	nd	2	aq, he
Opisthokonta			
Animalia			
Arthropoda	nd	15	aq, he, ps
Bilateria	nd	1	aq, so
Chordata	nd	1	he
Cnidaria	nd	1	he, ma, ps, so
Mollusca	nd	1	he, ma, ps, so
Rotifera	nd	1	aq, he, ps
Tardigrada	nd	1	he, ps, th
Fungi			
Ascomycota	nd	48	aq, he, ps, so
Basidiomycota	nd	29	aq, he, ps, so
Mucorales	nd	1	aa, aq, he
Rhizaria	nd	1	aq, he

EAST REGION OF EMBAYMENT (3,385 m)

TAXON	READS	UNIQUE SEQs.	CHARACTERS[a]
**BACTERIA**			
Acidobacteria	1	1	ac, aq, he, so
Actinobacteria	16,458	246	aq, al, de, ha, he, ma, nf, no, ps, so, th
Aquificae	1	1	an, aq, au, th
Bacteroidetes	65	10	al, an, aq, au, ps, so, th
Cyanobacteria	33	4	au, ps, th
Deinococcus/Thermus	9	3	ae, an, he, th
Firmicutes	610	12	ae, an, au, ha, ma, ps, th
Gemmatimonadetes	1	1	aq, so
Planctomycetes	1	1	an, aq, au, ma, nr
Proteobacteria			
Alphaproteobacteria	1754	100	aa, al, aq, au, ha, ma, me, nr, ps, sm, so, th
Betaproteobacteria.	2912	84	ae, aq, au, nf, me, no, nr, ps, am, th
Deltaproteobacteria	8	4	ae, an, aq, au, ha, ma, am, so
Gammaproteobacteria	2035	63	aa, ae, an, aq, au, ha, ic, ma, nf, no, nr, ps, sm, so, th
**ARCHAEA**	2	2	ha, he, ma, ps, so
**EUKARYA**			
Amoebozoa	5	5	aq, he
Archaeplastida			
Chlorophyta	1	1	au, ps
Streptophyta	93	47	au
Chromalveolata	3	3	aa, aq, au, ps, he
Ciliophora	2	2	aq, he

Opisthokonta			
Animalia			
Arthropoda	1	1	aq, he, ps
Choanoflagellates	1	1	aq, ma, he
Chordata	2	2	aq, ma, he, ps
Nematoda	1	1	aa
Platyhelminthes	1	1	aa
Fungi			
Ascomycota	109	22	aq, he, ps, so
Basidiomycota	21	2	aq, he, ps, so

MAIN BASIN (3,606 + 3,621 m)

TAXON	READS	UNIQUE SEQs.	CHARACTERS[a]
**BACTERIA**			
Actinobacteria	nd	14	aa, aq, he, ma, ps, so
Bacteroidetes/Chlorobi	nd	1	ae, al, he, ps
Chloroflexi	nd	1	ae, au, th
Deinococcus/Thermus	nd	1	he, th
Firmicutes	nd	16	aa, al, he, nr, ps, so, th
Fusobacteria	nd	1	aa, ae, he
Proteobacteria			
Alphaproteobacteria	nd	8	aa, ae, ac, aq, au, he, nf, ps, so
Betaproteobacteria	nd	22	aa, ae, aq, au, he, ma, nf, no, nr, ps, so, th
Gammaproteobacteria	nd	39	aa, ae, aq, au, ha, nf, no, nr, pa, ps, so, th

**EUKARYA**			
Archaeplastida			
Streptophyta	nd	2	au
Opisthokonta			
Animalia			
Arthropoda	nd	5	aa, aq, he
Fungi			
Ascomycota	nd	13	aq, he, ma, ps, th, so
Basidiomycota	nd	4	aq, he, ma, ps

[a] Character abbreviations: aa = animal associated; ac = acidopjhilic/acidotolerant; ae = aerobic; an = anaerobic; al- = alkaliphilic/alkalitolerant; aq = aquatic; au = autotrophic; de = desiccation-resistant; ha = halophilic/halotolerant; he = heterotrophic; ic = ice/polar associated; ma = marine; me = methane metabolizing; nf = nitrogen fixation; no = ammonia/nitrite/oxidation; nr = nitrite/nitrate reduction; ps = psychrophilic/psychrotolerant; sm = sulfur metabolism; so = soil/sediment inhabiting; th = thermophilic/thermotolerant;

Table S8. Number of Reads per Gene (gene category).

GLACIAL (2,149m)

Not determined (all uncultured cyanobacteria)

BASAL (3,501 + 3.520 m)

Gene Category	# Occurrences	Gene
ABC transporters	1	ABC transporter
	1	glycerol-3-phosphate ABC transporter ATP-binding protein
	1	Maltose/maltodextrin transport ATP-binding protein MalK
	1	phosphate ABC transporter
	1	sulfonate ABC transporter ATP-binding protein
ATPase	1	ATPase AAA-2 domain protein
	1	ATPase P
Amino acid metabolism	2	D-aminoacylase
	1	Saccharopine dehydrogenase
Cofactor synthesis	2	cobalamin biosynthesis protein CobN
	1	cobaltochelatase

	1	molybdopterin biosynthesis protein
DNA replication	2	ATP-dependent DNA helicase
	1	ATP-dependent DNA helicase II UvrD
Hydrolase	1	acylpyruvate hydrolase
Nucleotide metabolism	1	adenylate kinase
Oxidoreductase	1	betaine-aldehyde dehydrogenase
	1	gfo/Idh/MocA family oxidoreductase
	6	inositol 2-dehydrogenase
	5	oxidoreductase
Sulfur metabolism	1	homoserine O-succinyltransferase
Sugar metabolism	1	fructosamine kinase
	1	sugar-binding protein
Transferase	8	acetyl-CoA acetyltransferase
	2	CoA-transferase III family protein
Translation	1	ribosome biogenesis protein RLP24
Transport	1	cadmium-transporting ATPase
	1	cation-transporting ATPase G
	1	Vitamin B12 transport ATP-binding protein BacA
Other	1	ATP-binding protein
	15	Aim26p (Altered inheritance of mitochondrial protein 26)
	1	dehydrogenase
	5	hypothetical proteins
	4	non-ribosomal peptide synthetase
	1	polyphosphate kinase

	1	short-chain dehydrogenase/reductase SDR
	1	TetR family transcriptional regulator
	4	uncharacterized protein isoforms

WEST REGION OF EMBAYMENT (3540 + 3569 m)

Gene Category	# Occurrences	Gene
ABC transporters	5	ABC transporter
	1	ABC transporter ATP-binding protein
	1	carbohydrate ABC transporter ATP-binding protein
	2	glycerol-3-phosphate ABC transporter ATP-binding protein
	1	iron ABC transporter
	1	Maltose/maltodextrin transport ATP-binding protein MalK
	4	phosphate ABC transporter
	1	phosphate ABC transporter ATP-binding protein
	1	sulfonate ABC transporter ATP-binding protein
Amino acid metabolism	1	3-phosphoshikimate 1-carboxyvinyltransferase
	1	betaine-aldehyde dehydrogenase
	1	D-alanine aminotransferase
	1	prephenate dehydratase
	1	shikimate dehydrogenase
Cell structure	1	ankyrin
Chaperone	1	co-chaperone YbbN
Fatty acid metabolism	1	long-chain-fatty-acid--CoA ligase

Hydrolase	2	formyltetrahydrofolate deformylase
Nitrogen metabolism	1	NarG (nitrogen reductase)
	2	nitrate reductase
	1	nitrate reductase A subunit alpha
	7	nitrate reductase subunit alpha
	3	respiratory nitrate reductase alpha chain
	2	respiratory nitrate reductase alpha subunit apoprotein
Oxidoreductase	1	FMN-dependent dehydrogenase
Protease	1	CAAX protease self-immunity
Sugar metabolism	1	fructosamine kinase
Sulfur metabolism	1	homoserine O-succinyltransferase
Transferase	7	acetyl-CoA acetyltransferase
	1	CDP-glycerol--glycerophosphate glycerophosphotransferase
Translation	13	translational GTPase TypA
Transport	3	allantoin permease
	1	hexose transporter-like protein
	1	vitamin B12 transport ATP-binding protein BacA
	1	zinc transporter 10-like isoform X1
Other	1	amidohydrolase
	1	AMP-dependent synthetase
	2	group 1 glycosyltransferase
	6	GTP-binding protein
	11	GTP-binding protein TypA
	12	GTP-binding protein TypA/BipA
	7	histone deacetylase 11 isoform

	12	hypothetical proteins
	1	LVIVD repeat-containing protein
	53	phage minor tail protein
	1	peptide synthetase
	1	PRA1 family protein F3-like
	1	protein kinase
	1	sugar-binding protein
	1	phage tail protein
	3	thrombospondin-4
	1	transcript variant X4 (RNA splicing factor)
	1	tyrosine phosphorylated protein A
	3	uncharacterized
	4	uncharacterized protein isoforms

MIDDLE REGION OF EMBAYMENT (3,563 + 3,585 m)

Category	# Occurrences	Gene
ABC transporters	1	ABC transport system ATP-binding protein
	1	antibiotic transport system ATP-binding protein
	1	antibiotic transport system permease protein
	1	ATP-binding cassette, subfamily B, bacterial MsbA
	1	ATP-binding cassette, subfamily C, bacterial
	1	bacitracin transport system permease protein
	1	bicarbonate transport system ATP-binding protein
	2	branched-chain amino acid transport system ATP-binding protein
	2	cobalt/nickel transport system ATP-binding protein
	1	dipeptide transport system permease protein

	1	dipeptide transport system substrate-binding protein
	1	glutamine transport system substrate-binding protein
	1	histidine transport system ATP-binding protein
	1	iron complex transport system ATP-binding protein
	2	iron complex transport system permease protein
	1	iron(III) transport system ATP-binding protein
	1	lipoprotein-releasing system permease protein
	1	lipopolysaccharide transport system permease protein
	1	manganese/zinc/iron transport system ATP- binding protein
	1	multiple sugar transport system permease protein
	1	peptide/nickel transport system substrate-binding protein
	1	phosphonate transport system ATP-binding protein
	1	polar amino acid ABC transporter inner membrane subunit
	1	putrescine transport system substrate-binding protein
	1	ribose transport system ATP-binding protein
	1	ribose transport system permease protein
	2	sorbitol/mannitol transport system permease protein
	2	spermidine/putrescine transport system permease protein
	1	spermidine/putrescine transport system substrate-binding protein
	1	sulfate transport system ATP-binding protein
	1	sulfate transport system permease protein
	1	urea transport system substrate-binding protein
ATPase	1	arsenite-transporting ATPase
	2	Cu2+-exporting ATPase
Aminoacyl tRNA synthetase	1	arginyl-tRNA synthetase
	1	aspartyl-tRNA synthetase
	1	choline dehydrogenase
	1	cysteinyl-tRNA synthetase
	1	glycyl-tRNA synthetase beta chain
	3	histidyl-tRNA synthetase
	2	lysyl-tRNA synthetase, class I
	1	lysyl-tRNA synthetase, class II

	1	methionyl-tRNA synthetase
	2	phenylalanyl-tRNA synthetase alpha chain
	2	phenylalanyl-tRNA synthetase beta chain
	1	prolyl-tRNA synthetase
	1	tyrosyl-tRNA synthetase
	1	valyl-tRNA synthetase
Amino acid metabolism	1	acetolactate synthase I/II/III large subunit
	1	acetylornithine deacetylase
	1	agmatine deiminase
	2	alanine dehydrogenase
	1	alanine-glyoxylate transaminase
	1	alanine racemase, N-terminal
	1	amino-acid N-acetyltransferase
	1	argininosuccinate lyase
	1	asparagine synthase
	1	aspartate aminotransferase
	1	aspartate carbamoyltransferase catalytic subunit
	1	aspartate 4-decarboxylase
	1	aspartate-semialdehyde dehydrogenase
	1	ATP phosphoribosyltransferase
	1	branched-chain amino acid aminotransferase
	1	choline dehydrogenase
	1	cyclase
	2	cysteine synthase A
	1	D-alanine-D-alanine ligase
	1	D-cysteine desulfhydrase
	1	3-dehydroquinate synthase
	1	endo-1,4-beta-xylanase
	1	glutaminase
	2	glutamine amidotransferase
	2	glutamine synthetase
	1	glutamate racemase
	4	glycine dehydrogenase

	1	hippurate hydrolase
	1	histidinol-phosphate aminotransferase
	1	histidinol dehydrogenase
	1	homoserine kinase
	2	3-hydroxyisobutyrate dehydrogenase
	1	3-isopropylmalate/(R)-2-methylmalate dehydratase large subunit
	3	ketol-acid reductoisomerase
	3	L-aspartate oxidase
	1	methylmalonate-semialdehyde dehydrogenase
	1	monoamine oxidase
	1	ornithine carbamoyltransferase
	1	oxaloacetate decarboxylase, beta subunit
	2	pantoate--beta-alanine ligase
	1	peptidylprolyl isomerase
	1	3-(3-hydroxy-phenyl)propionate hydroxylase
	1	D-3-phosphoglycerate dehydrogenase
	1	prephenate dehydratase
	2	proline dehydrogenase /delta 1-pyrroline-5-carboxylate dehydrogenase
	1	proline iminopeptidase
	1	RNA polymerase nonessential primary-like sigma factor
	1	S-adenosylhomocysteine/5'-methylthioadenosine nucleosidase
	1	sarcosine oxidase, subunit alpha
	1	shikimate dehydrogenase
	1	2,3,4,5-tetrahydropyridine-2-carboxylate N-succinyltransferas
	1	tryptophan synthase beta chain
Butanoate metabolism	2	acetoacetyl-CoA synthetase
	1	formate C-acetyltransferase
Calcium signaling	1	Ca2+-transporting ATPase
	1	phosphorylase kinase alpha/beta subunit
Carbon fixation	1	3-hydroxyacyl-CoA dehydrogenase

Cell cycle	1	ATP-dependent Clp protease ATP-binding subunit ClpX
	1	ATP-dependent Clp protease, protease subunit
	1	ATP-dependent Lon protease
	1	glucose repression regulatory protein TUP1
	1	regulator of sigma E protease
Cofactor synthesis	1	4-hydroxythreonine-4-phosphate dehydrogenase
	1	molybdenum cofactor biosynthesis protein
	1	thiamine biosynthesis protein ThiC
	1	thiamine-phosphate pyrophosphorylase
DNA Recombination	1	ATP-dependent DNA helicase RecG
	1	deoxyribodipyrimidine photo-lyase
	1	holliday junction DNA helicase RuvB
	1	methylated-DNA-[protein]-cysteine S-methyltransferase
	2	primosomal protein N' (replication factor Y) (superfamily II helicase)
	1	recombination protein RecA
DNA repair	1	DNA helicase II / ATP-dependent DNA helicase PcrA
	1	DNA mismatch repair protein MutL
	1	DNA polymerase I
	1	DNA polymerase V
	1	excinuclease ABC subunit C
	1	excinuclease ABC subunit B
	1	exodeoxyribonuclease III
	1	exodeoxyribonuclease VII large subunit
	1	transcription-repair coupling factor (superfamily II helicase
DNA replication	1	ATP-dependent DNA helicase RecQ
	1	DNA gyrase subunit A
	3	replicative DNA helicase
	1	single-strand DNA-binding protein
Fatty acid metabolism	1	acyl-CoA dehydrogenase

	2	enoyl-[acyl carrier protein] reductase I
	1	enoyl-[acyl-carrier protein] reductase II
	1	enoyl-CoA hydratase
	2	fatty-acyl-CoA synthase
	4	3-hydroxydecanoyl-[acyl-carrier-protein] dehydratase
	1	3R-hydroxymyristoyl ACP dehydrase
	3	long-chain acyl-CoA synthetase
	1	omega-6 fatty acid desaturase (delta-12 desaturase)
	1	3-oxoacyl-[acyl-carrier-protein] synthase III
Glutathione metabolism	3	glutathione-independent formaldehyde dehydrogenase
Glycolysis-Gluconeogenesis	2	acetyl-CoA synthetase
	3	aldehyde dehydrogenase (NAD+)
	1	enolase
	1	glucose-6-phosphate isomerase
	6	glyceraldehyde 3-phosphate dehydrogenase
	1	phosphoenolpyruvate carboxykinase (ATP)
	2	pyruvate dehydrogenase E1 component
	1	pyruvate dehydrogenase E2 component (dihydrolipoamide acetyltransferase)
Hydrolase	1	haloalkane dehalogenase
Nitrogen metabolism	1	asparagine synthase (glutamine-hydrolysing)
	1	ferredoxin-nitrate reductase
	1	FMN-dependent NADH-azoreductase
	2	nitrate reductase 1, alpha subunit
	2	sulfonate/nitrate/taurine transport system permease protein
	1	ubiquinol-cytochrome c reductase cytochrome b subunit
Nucleotide metabolism	2	adenylate cyclase
	1	adenylosuccinate synthase
	1	altronate hydrolase
	1	amidophosphoribosyltransferase

1	exopolyphosphatase / guanosine-5'-triphosphate,3'-diphosphate pyrophosphatase
1	carbamoyl-phosphate synthase large subunit
1	2',3'-cyclic-nucleotide 2'-phosphodiesterase
1	cytosine deaminase
2	dCTP deaminase
1	CTP synthase
1	1-deoxy-D-xylulose-5-phosphate synthase
1	dihydroorotate dehydrogenase (fumarate)
1	DNA (cytosine-5-)-methyltransferase
1	DNA-directed RNA polymerase subunit beta
1	DNA polymerase III subunit delta
1	DNA polymerase III subunit epsilon
1	DNA polymerase III subunit gamma/tau
1	galactokinase
1	glucose-1-phosphate adenylyltransferase
2	GMP synthase (glutamine-hydrolysing)
1	GTP pyrophosphokinase
1	guanosine-3',5'-bis(diphosphate) 3'-pyrophosphohydrolase
1	IMP dehydrogenase
1	mannose-1-phosphate guanylyltransferase
1	N-acetylglucosamine-6-phosphate deacetylase
1	N-acetylneuraminate synthase
1	nucleoside-triphosphate pyrophosphatase
1	phosphoribosylamine--glycine ligase
1	phosphoribosylformylglycinamidine cyclo-ligase
1	phosphoribosylformylglycinamidine synthase
1	polyribonucleotide nucleotidyltransferase
1	pyrimidine operon attenuation protein / uracil phosphoribosyltransferase
1	pyrimidine-nucleoside phosphorylase
1	ribonucleoside-diphosphate reductase alpha chain
1	RNA-directed DNA polymerase
1	thymidine kinase
1	thymidylate synthase
1	UDP-galactopyranose mutase

	3	UDPglucose 6-dehydrogenase
	1	UDP glucose--hexose-1-phosphate uridylyltransferase
	1	UDP-N-acetylmuramoylalanine--D-glutamate ligase
	1	UDP-N-acetylmuramate dehydrogenase
	1	UDP-N-acetylglucosamine 2-epimerase
	1	UDP-N-acetylglucosamine pyrophosphorylase
	1	ureidoglycolate dehydrogenase
	1	ureidoglycine aminohydrolase
	1	xylulokinase
Oxidative phosphorylation	2	cytochrome bd-I oxidase subunit I
	1	cytochrome o ubiquinol oxidase subunit I
	1	F-type H+-transporting ATPase subunit alpha
	1	F-type H+-transporting ATPase subunit gamma
	1	glycerol-3-phosphate dehydrogenase
	1	H+-transporting ATPase
	1	inorganic pyrophosphatase
	7	NADH dehydrogenase
	1	succinate dehydrogenase iron-sulfur protein
	1	ubiquinol-cytochrome c reductase cytochrome b subunit
Oxidoreductase	1	chloride peroxidase
	1	cytochrome c peroxidase
	1	glycerol dehydrogenase
	1	molybdopterin oxidoreductase, iron-sulfur binding subunit
	1	NADH oxidase
Pentose phosphate pathway	1	2-dehydro-3-deoxygluconokinase
	1	ribose-phosphate pyrophosphokinase
	1	transaldolase
Peptidase	1	methionyl aminopeptidase
	1	tripeptide aminopeptidase

Photosynthesis	1	apocytochrome f
	1	coproporphyrinogen III oxidase
	1	glutamate-1-semialdehyde 2,1-aminomutase
	1	heme oxygenase
	1	light-independent protochlorophyllide reductase subunit B
	1	light-independent protochlorophyllide reductase subunit L
	1	magnesium chelatase subunit H
	1	nicotinate-nucleotide--dimethylbenzimidazole phosphoribosyltransferase
	1	phosphoribulokinase
	1	photosystem I P700 chlorophyll a apoprotein A1
	1	photosystem I P700 chlorophyll a apoprotein A2
	1	photosystem I subunit XI
	1	photosystem II CP43 chlorophyll apoprotein
	1	photosystem II P680 reaction center D1 protein
	1	phycocyanin beta chain
	1	precorrin-8X methylmutase
	2	pyruvate-flavodoxin oxidoreductase
	1	S-layer domain-containing protein
	1	uroporphyrinogen decarboxylase
RNA metabolism	4	ATP-dependent RNA helicase DeaD
	1	(di)nucleoside polyphosphate hydrolase
	1	exoribonuclease II
Secretion system	1	preprotein translocase subunit SecE
	1	preprotein translocase subunit SecY
	1	type IV secretion system protein VirB4
	1	type IV secretion system protein VirB9
Sulfur metabolism	1	sulfite reductase (NADPH) hemoprotein beta-component
	1	tRNA-specific 2-thiouridylase
TCA cycle	1	fumarate hydratase, class I
	2	isocitrate dehydrogenase

	2	2-oxoglutarate dehydrogenase (dihydrolipoamide succinyltransferase)
Transferase	1	aminoglycoside 3'-phosphotransferase
	1	glutamate-ammonia-ligase adenylyltransferas
	1	lipid-A-disaccharide synthase
	1	leucyl/phenylalanyl-tRNA--protein transferase
	1	ribosomal-protein-alanine N-acetyltransferase
	1	serine/threonine-protein kinase HipA
	1	UDP-N-acetylglucosamine acyltransferase
Translation	1	elongation factor 2
	2	elongation factor Tu
Transport	1	elongation factor 1-alpha
	1	LPS-assembly protein
	1	mechanosensitive ion channel
	1	MFS transporter, FSR family, fosmidomycin resistance protein
	1	preprotein translocase subunit SecY
Other	1	alpha-L-fucosidase
	1	alpha-galactosidase
	1	alpha-glucuronidase
	1	4-aminobutyrate aminotransferase / (S)-3-amino-2-methylpropionate transaminase
	1	anthraniloyl-CoA monooxygenase
	1	arylsulfatase A
	1	arachidonate 5-lipoxygenase
	1	beta-fructofuranosidase
	1	beta-galactosidase
	1	1,3-beta-glucan synthase
	1	beta-glucosidase
	1	beta-lactamase
	1	catalase
	1	cell division protein FtsI (penicillin-binding protein 3)

1	chromosomal replication initiator protein
1	CoA-binding protein
1	Cu(I)/Ag(I) efflux system membrane protein CusA
1	Cu(I)/Ag(I) efflux system membrane protein CusB
1	Cyan7425_1370 Signal transduction histidine kinase
1	dephospho-CoA kinase
1	dolichol-phosphate mannosyltransferase
1	4-diphosphocytidyl-2-C-methyl-D-erythritol kinase
1	endonuclease G, mitochondrial
1	Fe-S cluster assembly protein
2	formate dehydrogenase, alpha subunit
1	formate dehydrogenase, beta subunit
1	S-formylglutathione hydrolase
1	glutamate--cysteine ligase
1	glycolate oxidase FAD binding subunit
2	gamma-glutamyltranspeptidase
1	hemolysin D
1	heptaprenyl diphosphate synthase
1	3-hydroxybutyryl-CoA dehydratase
1	4-hydroxy-3-methylbut-2-enyl diphosphate reductase
1	glutathione S-transferase
1	lactoylglutathione lyase
1	L-ascorbate 6-phosphate lactonase
1	L-ribulose-5-phosphate 3-epimerase
1	leucyl aminopeptidase
1	lipid A biosynthesis lauroyl acyltransferase
1	malate dehydrogenase (oxaloacetate-decarboxylating)
1	maltose alpha-D-glucosyltransferase
1	membrane dipeptidase
1	methylenetetrahydrofolate dehydrogenase(NAD+) / 5,10-methenyltetrahydrofolate cyclohydrolase
1	NAD+ synthase (glutamine-hydrolysing)
1	oligosaccharyltransferase complex subunit beta
1	3-oxoadipyl-CoA thiolase

2	penicillin-binding protein
1	peptidyl-dipeptidase Dcp
1	phospholipase A-2-activating protein
1	phosphatidate cytidylyltransferase
1	phosphopantothenoylcysteine decarboxylase
1	[protein-PII] uridylyltransferase
1	propionyl-CoA carboxylase beta chain
1	protein phosphatase
1	PTS system, mannose-specific IIA component
2	PTS system, mannose-specific IIB component
1	pullulanase
1	pyruvate oxidase
1	pyruvate, water dikinase
1	repressor LexA
1	signal peptidase II
1	starch synthase
1	sucrose phosphorylase
1	2-succinyl-5-enolpyruvyl-6-hydroxy-3-cyclohexene-1-carboxylate synthase
1	superoxide dismutase, Fe-Mn family
1	tartronate-semialdehyde synthase
1	dTDP-glucose 4,6-dehydratase
2	dTDP-4-dehydrorhamnose 3,5-epimerase
1	trans-aconitate 2-methyltransferase
1`	transcription termination factor Rho
2	transposase
1	tRNA dimethylallyltransferase
1	two-component system, cell cycle response regulator
1	two-component system, chemotaxis family, CheB/CheR fusion protein
1	two-component system, chemotaxis family, sensor kinase CheA
1	two-component system, NarL family, sensor histidine kinase DesK
1	two-component system, NtrC family, sensor histidine kinase HydH
1	two-component system, OmpR family, heavy metal sensor histidine kinase CusS
1	two-component system, OmpR family, osmolarity sensor histidine kinase EnvZ
1	two-component system, OmpR family, phosphate regulon sensor histidine kinase

		PhoR
	1	two-component system, OmpR family, response regulator VicR
	1	type I restriction enzyme, R subunit
	1	3D-(3,5/4)-trihydroxycyclohexane-1,2-dione hydrolase
	1	ubiquinone/menaquinone biosynthesis methyltransferase
	1	UDP-3-O-[3-hydroxymyristoyl] N-acetylglucosamine deacetylase
	1	UDP-N-acetylglucosamine--N-acetylmuramyl-(pentapeptide) pyrophosphoryl-undecaprenol N-acetylglucosamine transferase
	2	UDP-N-acetylmuramoylalanyl-D-glutamyl-2,6-diaminopimelate--D-alanyl-D-alanine ligase
	2	undecaprenyl-diphosphatase synthase
	1	zinc metalloprotease

EAST REGION OF EMBAYMENT (3,385 m)

Category	# Occurrences	Gene
ABC transporters	1	ABC transporter
	1	ABC-type antimicrobial peptide transport system
ATPase	1	AtpE
	11	ATP synthase CF1 epsilon subunit
	1	ClpV1 family T6SS ATPase
Amino acid metabolism	5	cobalamin synthesis protein
	3	D-aminoacylase
	14	gamma-glutamyl phosphate reductase
	11	glutamate-5-semialdehyde dehydrogenase

	2	glycine decarboxylase
	26	imidazole glycerol phosphate synthase
	1	methionine synthase (B12-independent)
	2	5-methyltetrahydropteroyltriglutamate-homocysteine methyltransferase
	1	phosphoserine aminotransferase
	1	phosphoserine transaminase
	1	poly(enriched lactate-co-3-hydroxybutyrate) depolymerase
	1	prephenate dehydratase
Chaperone	1	co-chaperone YbbN
	2	metal chaperone
Cofactor synthesis	16	cobalamin biosynthesis protein
	2	cobW/HypB/UreG
	1	2-succinylbenzoate--CoA ligase
DNA repair	3	excinuclease ABC
	8	excinuclease ABC subunit A
	2	excinuclease ABC subunit UvrA
DNA replication	7	ATP-dependent helicase
Fatty acid metabolism	5	acetyl/propionyl-CoA carboxylase subunit alpha
	1	acetyl-CoA carboxylase
	1	acetyl-CoA carboxylase biotin carboxylase subunit
	1	acyl-CoA synthetase
	4	acyl-CoA synthetase (AMP-forming)/AMP-acid ligase II
	7	AMP-dependent synthetase
	1	Fatty-acid-CoA ligase FadD7
	1	fatty-acyl-CoA synthase
	2	long-chain fatty acid--CoA ligase
Glycolysis-Gluconeogenesis	1	3-phosphoglycerate kinase
	62	phosphoglycerate kinase

Nitrogen metabolism	1	nitrile hydratase activator
Nucleotide metabolism	2	carbamoyl-phosphate synthase L chain ATP-binding
	1	GTP pyrophosphokinase
	19	thymidylate synthase protein
Oxidoreductase	3	FAD dependent oxidoreductase
	2	Glucose-methanol-choline (GMC) oxidoreductase:NAD binding site
	2	GMC family oxidoreductase
	2	2-keto-gluconate dehydrogenase
Ribonuclease	1	NYN domain
	1	polyribonucleotide nucleotidyltransferase
Sugar/carbohydrate metabolism	1	beta-glucosidase
	1	fructose-1
	1	fructosamine kinase
	1	fructose dehydrogenase large subunit
	2	glucoamylase
	1	glycogen branching enzyme
	2	group 1 glycosyltransferase
Sulfur metabolism	1	sulfatase
TCA cycle	5	propionyl-CoA carboxylase
Translation	1	50S ribosomal protein L21
	31	elongation factor 4
	1	translation elongation factor LepA
	8	translational GTPase TypA
	3	GTP-binding elongation factor
	21	GTP-binding protein LepA
	28	GTP-binding protein TypA

	1	Translational regulator
Transport	7	acetyl-CoA acetyltransferase
	1	cadmium transporter
	6	cation transporter
	2	cobalt-zinc-cadmium resistance protein CzcA
	7	CusA/CzcA family heavy metal efflux RND transporter
	1	efflux transporter periplasmic adaptor subunit
	1	heavy metal efflux pump
	1	hexose transporter-like protein
	4	preprotein translocase subunit SecF
	2	zinc transporter 10-like isoforma
Other	1	ATP/GTP-binding protein
	11	ATP-dependent RNA helicases HrpA/HrpB
	1	Bifunctional (p)ppGpp synthase/hydrolase relA
	1	biotin carboxyl carrier protein
	1	COBW domain-containing protein
	1	glutathione S-transferase Mu 2
	9	glycine dehydrogenase
	1	GTPase
	1	GTP-binding protein
	3	GTP pyrophosphokinase
	10	lysine-specific demethylase PHF2
	6	4-hydroxytetrahydrobiopterin dehydratase
	35	hypothetical protein
	1	keratinocyte proline-rich protein
	1	LVIVD repeat-containing protein
	1	modular polyketide synthase
	1	N-lysine methyltransferase SETD8-A-like
	1	O-succinylbenzoate-CoA ligase
	4	PHD finger protein 2
	1	PRA1 family protein F3-like
	1	RNA helicase

	1	sorbosone dehydrogenase
	1	sugar-binding protein
	1	TetR family transcriptional regulator
	1	transmembrane protein 151B
	1	type IV secretory pathway
	1	type VI secretion ATPase
	2	UDP-glucose 6-dehydrogenase UdgA
	1	UDP-glucose glycoprotein glucosyltransferase 1
	1	UDP-glucose/GDP-mannose dehydrogenase family protein
	1	YihY family inner membrane domain protein

MAIN BASIN (3,606 + 3,621 m)

Category	# Occurrences	Gene
Amino acid metabolism	1	aspartate kinase
	1	LysR family transcriptional regulator
	1	glutamate--cysteine ligase
	1	glutamine amidotransferase
Citrate cycle	1	succinyl-CoA synthetase beta subunit
DNase	1	type I restriction enzyme, S subunit
DNA repair	1	excinuclease ABC subunit B
Nucleotide metabolism	1	cytosine deaminase
	1	UDP-N-acetylglucosamine 2-epimerase
Oxidative phosphorylation	2	cytochrome c oxidase subunit II

	1	cytochrome c oxidase assembly protein subunit 15
	1	cytochrome o ubiquinol oxidase subunit I
Pentose phosphate pathway	1	transketolase
Protease	1	ATP-dependent Clp protease adaptor protein ClpS
Transferase	1	colanic acid biosynthesis acetyltransferase
	1	phosphotransferase system, enzyme I, PtsI
Translation	1	GTPase (translation)
Transport	1	Cu2+-exporting ATPase
	1	multidrug efflux transporter MdtA
Transposase	1	Transposase
Other	1	chitin-binding protein
